# Versatile electronic phases enabled by intertwined multiple frustrations in an antiferromagnetic two-dimensional semimetal

**DOI:** 10.1038/s41467-026-75048-y

**Published:** 2026-07-23

**Authors:** Y. Fujisawa, P. Wu, T. Nakamura, R. Okuma, T. Kato, B. R. M. Smith, D. Ueta, R. Kobayashi, N. Maekawa, C-H. Hsu, C. De, N. Tomoda, T. Higashihara, K. Morishita, K. Sumida, K. Miyamoto, T. Okuda, Z. Y. Wang, Y. Okada

**Affiliations:** 1 Quantum Materials Science Unit, Okinawa Institute of Science and Technology (OIST), Okinawa, Japan; 2 Research Institute for Synchrotron Radiation Science (HiSOR), Hiroshima University, Higashi-Hiroshima, Japan; 3 Department of Physics and Chinese Academy of Sciences Key Laboratory of Strongly-coupled Quantum Matter Physics, University of Science and Technology of China, Hefei, Anhui China; 4 Institute for Solid State Physics (ISSP), The University of Tokyo, Kashiwa, Chiba, Japan; 5 JST, PRESTO, 4-1-8 Honcho, Kawaguchi, Saitama, Japan; 6 Institute of Materials Structure Science, High Energy Accelerator Research Organization, Tsukuba Ibaraki, Japan; 7 Faculty of Science, University of the Ryukyus, Nishihara, Okinawa, Japan; 8 Department of Physics, Graduate School of Science, Osaka University, Toyonaka, Japan; 9 International Institute for Sustainability with Knotted Chiral Meta Matter (WPI-SKCM2), 1-3-1 Kagamiyama, Higashi-Hiroshima, Japan; 10 Research Institute for Semiconductor Engineering (RISE), Hiroshima University, Higashi-Hiroshima, Japan

**Keywords:** Electronic properties and materials, Magnetic properties and materials, Two-dimensional materials

## Abstract

Following the discovery of graphene, van der Waals materials have become a major platform for quantum matter, yet strongly interacting phases beyond graphene remain elusive. Using scanning tunneling microscopy and spectroscopy, we uncover three competing stripe- and checkerboard-type multiple-q antiferromagnetic charge-ordered states in the van der Waals semimetal CeTe_3_, beyond the conventional charge density wave. These field-tunable states exhibit propagation vectors of (0.33, 0), (0, 0.08), and (0.19, ±0.19), and are controlled by a modest in-plane magnetic field (~1.5 T). Quasiparticle-interference imaging identifies corresponding three distinct nesting channels and associated Fermi-surface reconstructions. At the same time, spectroscopy reveals a broad electronic reconstruction extending to ±30 meV around the Fermi level, beyond a simple Kondo-coupling picture. Our results establish CeTe_3_ as a platform for tunable nanoscale antiferromagnetic electronic phases characterized by intertwined correlations, exotic symmetry-breaking, and nontrivial topology.

## Abstract (web summary)

Competing frustration channels in low-dimensional antiferromagnetic metals can generate diverse single-q and multi-q electronic phases. Here, the authors identify three competing antiferromagnetic charge-ordered states in CeTe_3_.

## Introduction

Fermi-surface (FS) reconstruction and pronounced reshaping of the density of states (DOS) near the Fermi level (*E*_F_) are hallmarks of the emergence of distinct electronic phases, often associated with spontaneous symmetry breaking and resulting in exotic electronic functionality. While nesting of low-dimensional Fermi surfaces provides a conventional route to FS instabilities, the phenomenology becomes markedly richer when fermionic systems are subject to strong frustration, in which one state is selected from many competing possibilities. In contrast to quasi-one-dimensional systems—where nearly perfect nesting typically stabilizes an insulating state characterized by single-**q** spontaneous electronic ordering—quasi-two-dimensional systems with large FSs intrinsically exhibit imperfect nesting, giving rise to a wide variety of competing FS instabilities ^1–4^. Furthermore, when frustrated Fermi-surface instabilities coexist with other frustration channels, such as lattice/charge/orbital/spin ordering, they can give rise to a variety of single-**q** and multi-**q** intertwined phases, ranging from fluctuating regimes to long-range ordered states, reminiscent of the rich phenomenology of strongly correlated systems such as high-temperature superconductors^5–9^. In particular, controlling magnetic phases characterized by multi-**q**, frustrated FS instabilities is of great interest for developing topological magnetism and associated exotic electronic transport arising from giant fictitious fields^10–16^.

In this context, such opportunities are particularly compelling in van der Waals (vdW) metals hosting frustrated antiferromagnetism (AF). Unlike **q** = 0 ferromagnets, AF states characterized by finite ordering wavevectors **q**_m_ can, in general, exhibit greater versatility. Furthermore, their interplay with competing nesting and frustrated FS instabilities can yield unexpected intertwined phases that go beyond the phenomenology of ubiquitous charge order in vdW metals^17–20^. Such complex frustration and successive phase transitions, which typically drive the system toward reduced carrier density and more localized electronic states, can recruit additional many-body interactions, giving rise to exotic strongly correlated phases accompanied by electronic reconstructions over unusually broad energy scales around *E*_F_, beyond weak-coupling descriptions^21^. However, how this interplay evolves across energy, momentum, and external control parameters remains poorly understood in antiferromagnetic vdW metals and semimetals, despite these promising prospects and the ubiquitous observation of charge-density-wave (CDW) order in many non-magnetic vdW materials^22^.

**Fig. 1:**
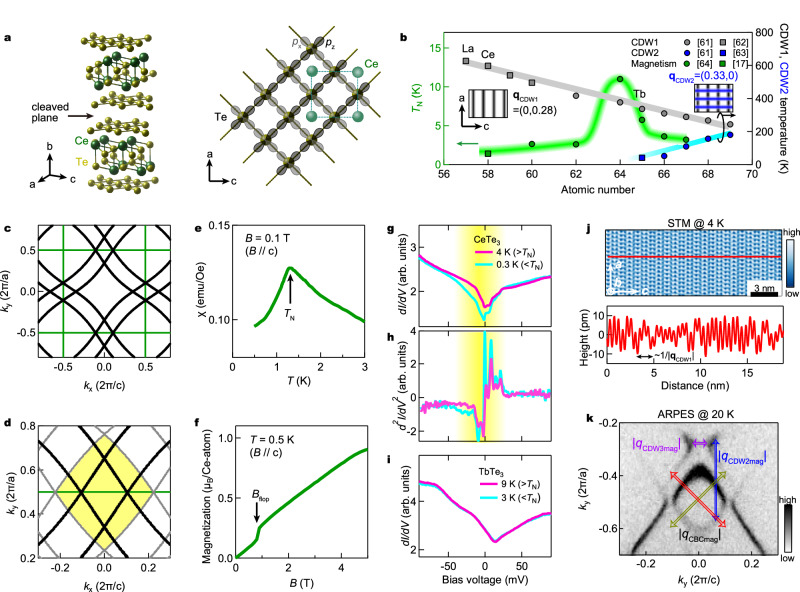
Unique position of CeTe_3_ among the RTe_3_ family. **a** Crystal structure of CeTe_3_ and top view of the lattice. The arrow in the left panel indicates the cleaved plane. **b** Schematic phase diagram of the RTe_3_ family, illustrating two well-established charge density wave (CDW) transitions (CDW1 and CDW2) and the Néel temperature (*T*_N_), based on ref. ^17,61–64^. Gray, cyan, and green curves represent guides to the eye for CDW1, CDW2, and antiferromagnetic transition temperatures. The inset black and blue modulation represent the CDW1 and CDW2, respectively. Simplified FS topology derived from a tight-binding model considering Te 5*p*ₓ and 5*p*ᵧ orbitals forming a square net and block layer band folding (**c**), and additional folding due to the CDW1 formation (**d**). Green lines represent the Brillouin zone for the unit cell. The focused FSs are shaded by yellow. Temperature (**e**) and magnetic field (**f**) dependence of the magnetization in CeTe_3_. Black arrows indicate magnetic transition points. The data is adopted from our previous study^17^. Comparison of the spatially averaged *dI/dV* spectra across *T*_N_ for CeTe_3_ (**g**), its derivative (**h**) and the spectra for TbTe_3_ (**i**), respectively. The set-point is 90 mV/2.5 nA with lock-in amplitude of 0.4 mV (961 Hz) for CeTe_3_ and -50 mV/400 pA with lock-in amplitude of 1 mV (961 Hz) for TbTe_3_. The characteristic energy window focused in this study is shaded by yellow. **j** The topographic image of CeTe_3_ at 4 K, representing CDW1 and a line profile along the red line. **k** Constant energy contour at the Fermi level obtained by ARPES at 20 K.

The rare-earth tri-telluride (RTe_3_) family (Fig. 1a) has emerged as a versatile vdW quantum material platform^23^. These materials offer tunability through axial structural control^24^, chemical substitution^17,25,26^, exfoliation^27–29^, and external stimuli such as pressure, pulsed light, and radio-frequency fields^30–34^. Also, interesting signatures of pressure-induced superconductivity^35–37^, the formation of Kramers nodal line band structures^38^, and possible links to a Higgs mode analogous in high-energy physics^39,40^ are shown. Across the family, the initial two bands formed from Te 5*p* orbitals on square-net layers are folded by the potential of the block layers, resulting in a relatively simple parent FS in the absence of any CDWs (Fig. 1c). On top of this, CDW1—which is common across all RTe_3_ compounds (Fig. 1b)—introduces additional band folding and associated frustrating quasi-nesting (Fig. 1d). Intriguingly, Landau-level spectroscopy in LaTe_3_ reveals well-defined electron–hole pockets emerging from this otherwise complex folded structure (see the yellow shaded area in Fig. 1d)^41^. Thus, the significance of considering the instability of the Fermi surface in such representative semimetals—characterized by coexisting relatively small electron and hole pockets—becomes effective. This simple viewpoint captures the essence of the parent electronic state that incorporates the CDW1 characteristics common to the RTe_3_ family and provides a crucial foundation for deepening our understanding of emergent low-temperature phases.

While early studies predominantly focused on high-temperature charge-ordered states (CDW1 and CDW2)^4^, recent efforts have highlighted intertwined orders that emerge within antiferromagnetic phases below the Néel temperature (*T*_N_)^42–45^. In this context, CeTe_3_ stands out as a singular member that enables a controlled entry point into intertwined charge–spin physics in two dimensions^17,46–49^. In CeTe_3_, a 4*f*^1^ moment develops long-range AF order at *T*_N_ ≈ 1.5 K and undergoes a spin-flop transition at *B*_flop_ ≈ 1.5 T by in-plane field along *c* direction, which reorients the in-plane Néel vector (Fig. 1e, f). Concomitantly, CeTe_3_ exhibits a pronounced and unusually broad spectral reconstruction across *T*_N_, extending the energy scale of approximately ± 30 meV from *E*_F_ (shaded region in Fig. 1g, h), a feature absent in other RTe_3_ such as TbTe_3_ (Fig. 1i). The fact that this magnetic transition manifests directly as a modification of the electronic states near *E*_F_ indicates an intimate coupling between magnetism and the low-energy electronic structure, likely arising from moderate Kondo coupling between the Ce 4*f*^1^ moments and Te 5*p* electrons^46^. This interpretation is consistent with previous specific-heat measurements, which indicate a moderately screened Ce moment relative to 1 *μ*_B_^17^. Further insight is provided by neutron-scattering experiments, which reveal a magnetic propagation vector **q**_m_ = (*h, l*) = (0.17, ±0.31) at *B* = 0^49^. This information provides a valuable reference for comparison with the charge-propagation vector **q**_c_, if determined, thereby enabling a deeper understanding of the underlying spin–charge coupling mechanism. Despite the promising opportunities offered by CeTe_3_, the microscopic origin of these effects—and how charge and magnetic orders are selected and coupled—remains unresolved, motivating local and momentum-resolved spectroscopic studies under controlled temperature and magnetic-field conditions.

Here, using spectroscopic scanning tunneling microscopy (STM), we track the evolution of the electronic structure of CeTe₃ across *T*_N_ and *B*_flop_ (Fig. 1e, f)^17^. On the established parent state hosting only CDW1 with **q**_CDW1_ = (0, 0.28) for *T* > *T*_N_ (Fig. 1j)^50–52^, we identify three additional charge modulations with wavevectors (0.33, 0), (0.19, ±0.19), and (0, 0.08) that emerge within the AF state, all of which approximately correspond to the distinct Fermi surface nesting vectors (Fig. 1k). All measurements employed a non-magnetic STM tip, enabling direct access to intrinsic DOS reconstructions across *T*_N_ and *B*_flop_ without convolution from spin-dependent tunneling matrix elements inherent to spin-polarized STM. By combining real-space STM with quasiparticle-interference (QPI) imaging, focusing on magnetically ordered states below *T*_N_, we demonstrate microscopic insights into versatile antiferromagnetic charge order and its competition in momentum space in CeTe_3_. Based on these insights, we discuss and provide a foundation for future efforts to control and exploit intertwined orders—and their field-tunable competition—applicable broadly to AF vdW materials.

## Results and Discussions

**Fig. 2:**
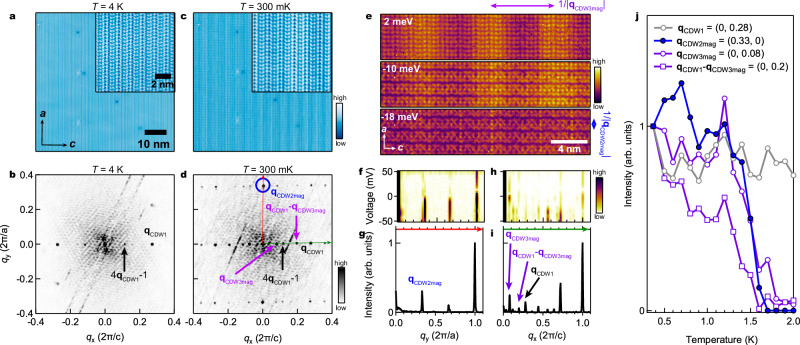
Temperature dependence of CDW2_mag_ and CDW3_mag_. The topographic image at 4 K (**a**) and its corresponding Fourier transform (FT) image (**b**). The topographic image at 300 mK (**c**), and its FT image (**d**). The setpoint conditions for (**a**) and (**c**) are similar (50 mV/4 nA and 50 mV/5 nA, respectively). All peaks corresponding in (**b**) and (**d**) are identified as a linear combination of relevant peaks of charge orders and crystal structures. See Supplementary Note 1 for the full assignment of the FFT components. **e** The *dI/dV* maps acquired over a 5 × 23 nm² area at various energies from −50 mV to 0 mV (setpoint conditions: 50 mV/5 nA with a lock-in modulation of 2 mV). The blue and purple arrows indicate the periodicity appeared only below *T*_N_ corresponding to **q**_CDW2mag_ and **q**_CDW3mag_, respectively. **f**, **h** The energy evolution of the line-cut profiles along *q*_y_ and *q*_x_ directions obtained from the *dI/dV* maps (Fig. 2e) (see the arrow indicated in (**d**)). **g**, **i** Line-cut profiles of the Fourier signal along the *q*_y_ and *q*_x_ direction at 300 mK. The signal is obtained from the topographic image (Fig. 2d). **j** Temperature dependence of the Fourier components at (0.33, 0), (0, 0.28), (0, 0.08) and (0, 0.2) extracted from the topographic images. The setpoint is −10 mV/500 pA.

We first identify magnetically driven charge orders from STM measurements performed across the AF transition at *T*_N_ ≈ 1.5 K under zero magnetic field (see Methods for sample characterization). At 4 K, the topographic image (Fig. 2a) exhibits a unidirectional modulation with a Fourier wavevector q_CDW1_ = (0, 0.28) (Fig. 2b), consistent with the well-established CDW1 phase of CeTe_3_ (Fig. 1b). Upon cooling to 300 mK, qualitatively distinct stripe-like modulations emerge. One is oriented perpendicular to CDW1 and is characterized by charge-propagation vectors (0.33, 0), and another is parallel to CDW1, characterized by (0, 0.08) (Fig. 2c, d). The same periodicities are clearly resolved in energy-dependent *dI/dV* maps in real space (Fig. 2e). They are consistently captured in Fourier space (Fig. 2f–i), visualizing their non-dispersive nature with energy. Because the wavevector (0.33, 0) resembles the conventional CDW2 reported in other RTe_3_ compounds, establishing whether this modulation is intrinsically linked to antiferromagnetism requires a detailed temperature-dependent study.

For efficient tracking of the relevant charge orders, we note that while the modulation at (0.33, 0) can be visualized over a relatively wide bias window of ±50 mV, topographic images acquired within a narrower bias window of ±10 mV are optimal for resolving the modulation with wavevector (0, 0.08) (see Methods and Supplementary Note 2). Accordingly, to track the simultaneous evolution of both charge orders, we focus on topographic images acquired, for example, at −10 mV. Notably, by following this strategy, while CDW1 shows little change across *T*_N_, the additional charge modulations characterized by wavevectors (0.33, 0) and (0, 0.08) collectively exhibit a clear onset below *T*_N_ (Fig. 2j). Combined with their suppression above the spin-flop field *B*_flop_ (discussed later), the modulation (0.33, 0) is established as a charge order intrinsically intertwined with antiferromagnetism, which we denote CDW2_mag_. Similarly, the modulation at (0, 0.08), which also develops exclusively below *T*_N_, is denoted CDW3_mag_ (Fig. 2d, j). Throughout this work, the subscript “mag” is used to indicate charge orders that emerge only within magnetically ordered states.

**Fig. 3:**
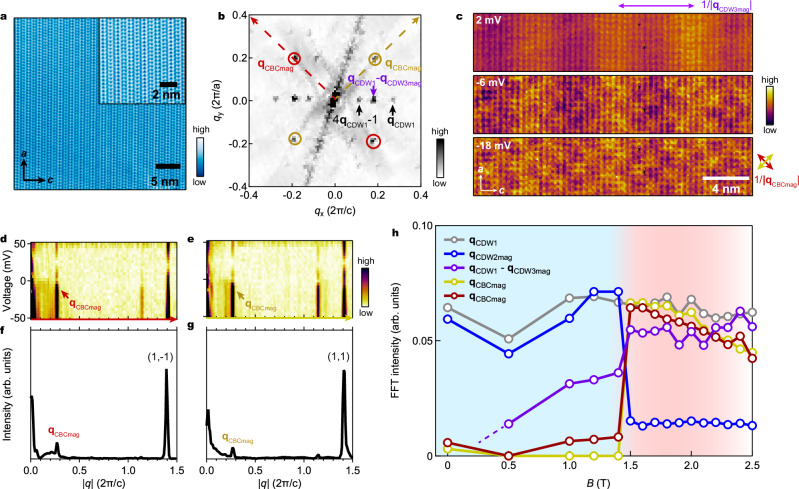
Competing CDW2_mag_ and CBC_mag_ at 300 mK across the spin-flop transition at B_flop_ under an in-plane magnetic field applied along the c-axis. **a** STM topograph acquired at *B* = 2 T applied along the *c*-axis at 300 mK. **b** FT image corresponding to (**a**). The measurement conditions for (**a**) are 50 mV/2.5 nA. See Supplementary Note 1 for the full assignment of the FFT components. **c** The *dI/dV* mapping at various energies as indicated inside. The set point is 50 mV/2.5 nA with the lock-in modulation of 2 mV. **d**, **e** The energy evolution of the line-cut profiles along (1,-1) and (1,1) directions, respectively (see the red and yellow arrows indicated in **b**). **f**, **g** Line-cut profiles of the Fourier signal of the STM topograph (**b**) along (1,-1) and (1,1) directions, respectively. **h** Magnetic field evolution of the FT peak intensities at **q**_CDW1_, **q**_CDW2mag_, **q**_CDW1_-**q**_CDW3mag_, and **q**_CBCmag_, which are normalized by the Bragg peaks at (*q*_y_,*q*_x_) = (0,1), (1,0), (0,1), (±1,1), respectively, extracted from topographic images taken with the set-point of +50 mV/2.5 nA.This choice of normalization factors for each charge modulation is not to compare the amplitude between them, but to reduce the effect of a small tip anisotropy. The background colors represent the magnetic phases across *B*_flop_.

Applying an in-plane magnetic field induces a pronounced reorganization of charge order within the AF phase. Similar to the previous study^17^, in-plane field is applied along horizontal c-axis, which is parallel to the CDW1 direction (Fig. 1f). At *B* = 2.0 T, exceeding the spin-flop field *B*_flop_ ≈ 1.5 T, the topographic image and its Fourier transform (Fig. 3a, b) reveal the emergence of two orthogonal charge modulations with wavevectors (0.19, ±0.19) (red and yellow circles in Fig. 3b). These modulations are directly visualized in real-space *dI/dV* maps (Fig. 3c) and corroborated by Fourier line profiles (Fig. 3d–g). The absence of measurable energy dispersion indicates that these modulations originate from static ordering (Fig. 3d, e). Because this charge order appears only within the magnetically ordered phase and is stabilized by an applied magnetic field, we refer to this state as CBC_mag_.

It should be noted that the charge-propagation vectors associated with CDW2_mag_, CDW3_mag_, and CBC_mag_ connect nearly nested regions of the Fermi surface (Fig. 1k). As a result, competition among these orders is naturally expected: stabilization of one charge order partially gaps the Fermi surface, thereby reducing the fermionic energy gain available to competing instabilities. Here, we use the term “nesting” in a broad sense, referring not only to ideal parallel Fermi-surface segments but also more generally to wavevectors connecting regions with a relatively large joint DOS. Within the scope of this study, deviations from ideal nesting conditions are not emphasized, provided that partial gap opening still yields a substantial net fermionic energy gain.

The relevant competition is directly visualized through the magnetic-field dependence of the Fourier intensities associated with CDW2_mag_, CDW3_mag_, and CBC_mag_ (Fig. 3h). While CDW3_mag_ is optimally visualized using bias voltages of approximately ±10 meV (Fig. 2c, d), we find that the competition between CDW2_mag_ and CBC_mag_ is more efficiently captured in topographic images acquired at larger bias voltages on the order of ±50 meV (see Methods and Supplementary Note 2). As shown in Fig. 3h, the CDW3_mag_-related signal is nearly buried within the background noise at a bias voltage of +50 meV at *B* = 0 (see dashed line). With increasing magnetic field, however, the enhancement of the CDW3_mag_ signal enables the simultaneous visualization of CDW2_mag_, CDW3_mag_, and CBC_mag_ within a single topographic dataset, allowing a consistent comparison across the entire field range. Here, to minimize extrinsic anisotropic contrast arising from the STM tip, the Fourier intensities are normalized by the corresponding Bragg peaks: (0,1) for CDW1 and CDW3_mag_, (1,0) for CDW2_mag_, and (1, ±1) for CBC_mag_. Although CDW3_mag_ evolves relatively smoothly with the magnetic field, a weak discontinuity is nevertheless observed across the spin-flop field *B*_flop_. More strikingly, as the magnetic field increases from zero, the intensity of CDW2_mag_ is sharply suppressed. At the same time, CBC_mag_ is enhanced across *B*_flop_, leading to a pronounced anti-correlation between the two orders.

**Fig. 4:**
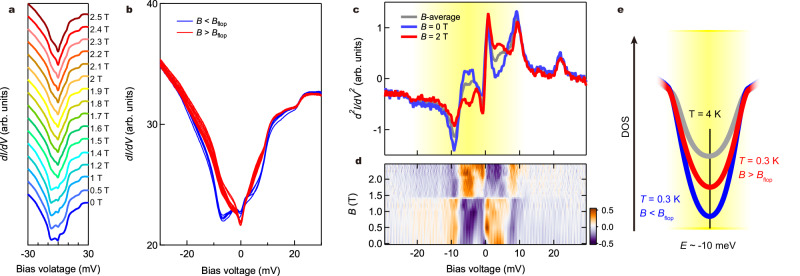
*dI/dV* deformation at 300 mK under an in-plane magnetic field applied along the c-axis. **a** Evolution of the spatially averaged *dI/dV* spectra under in-plane magnetic fields at 300 mK. Spectra are vertically offset for clarity. The measurement condition is 50 mV/2.5 nA with the lock-in modulation of 0.8 mV. **b** Representative spectra above and below the spin-flop field (*B* > *B*_flop_ and *B* < *B*_flop_), highlighted in two distinct colors. **c** Derivative of the *dI/dV* spectra (*d*^2^*I*/*dV*^2^) at *B* = 0 and 2 T. The yellow-highlighted region indicates the energy window where substantial spectral renormalization is observed. The gray line indicates the magnetic field averaged spectrum (*d*^2^*I/dV*^2^_B-ave_, obtained by averaging six curves (0, 0.5,1, 1.5, 2, 2.5 T), where three are representative for *B* < *B*_flop_ (0, 0.5, 1 T) and the other three are representative for *B* > *B*_flop_ (1.5, 2.0, 2.5 T). **d** The Magnetic field dependence of *d*^2^*I*/*dV*^2^(*B*)-*d*^2^*I/dV*^2^_B-ave_, revealing the pronounced spectral evolution within −20 meV to +10 meV. **e** Schematic spectral evolution with changing temperature and magnetic field.

The energy scale associated with the competition among multiple magnetic charge-ordered states is directly reflected in the magnetic-field dependence of the tunneling spectra *dI/dV*(*E*) (Fig. 4a). Using the bimodal color scheme (blue and red) shown in Fig. 4b, the spectra can be clearly classified into two distinct groups below and above the spin-flop field *B*_flop_. To quantify the characteristic energy scale, we directly compare representative spectra measured at *B* = 0 T (<*B*_flop_) and *B* = 2 T (> *B*_flop_). Taking the derivatives of the *dI/dV* spectra and overlaying them (Fig. 4c) highlights the energy range over which the DOS is most strongly modified. The gray curve in Fig. 4c represents a reference spectrum, denoted *d*^2^*I/dV*^2^_B-ave_, obtained by averaging representative spectra shown in Fig. 4a and b. This comparison demonstrates that the dominant spectral changes are confined to an energy window of approximately ±20 meV around *E*_F_. Consistently, the field evolution, plotted as *d*^2^*I/dV*^2^(*B*)−*d*^2^*I/dV*^2^_B-ave_, further reveals a pronounced DOS reconstruction across *B*_flop_ within the same energy range (Fig. 4d).

Rather than emphasizing fine spectral structures visible in Fig. 4d, the most robust outcome of the magnetic-field-dependent analysis is the identification of this characteristic energy window. In two-dimensional multiband systems, superlattice formation driven by Fermi-surface nesting typically produces complicated fine spectral features within the reconstructed energy range. Moreover, in LaTe_3_—where only CDW1 order exists (Fig. 1b)—electron–boson coupling generates dip-like structures near *E*_F_ that are not directly related to the CDW condensation energy^41^. Similar spectral complexity is therefore expected in CeTe_3_. In this context, the energy scale of the DOS modulation across *B*_flop_ provides a more reliable indicator than the detailed fine structure within the soft gap.

Combining the field-dependent evolution of the raw spectra (Fig. 4b) with the derivative analysis yields a consistent picture: the electronic structure changes across *B*_flop_ on an energy scale of approximately ±20 meV. Notably, the dominant spectral modifications extend across E_F_, indicating that the DOS reconstruction reflects a fermionic energy gain or loss associated with the competing phases. As schematically illustrated in Fig. 4e, the strongest manifestation of this energy competition appears near −10 meV, highlighted by the shaded regions in Fig. 4b and c.

**Fig. 5:**
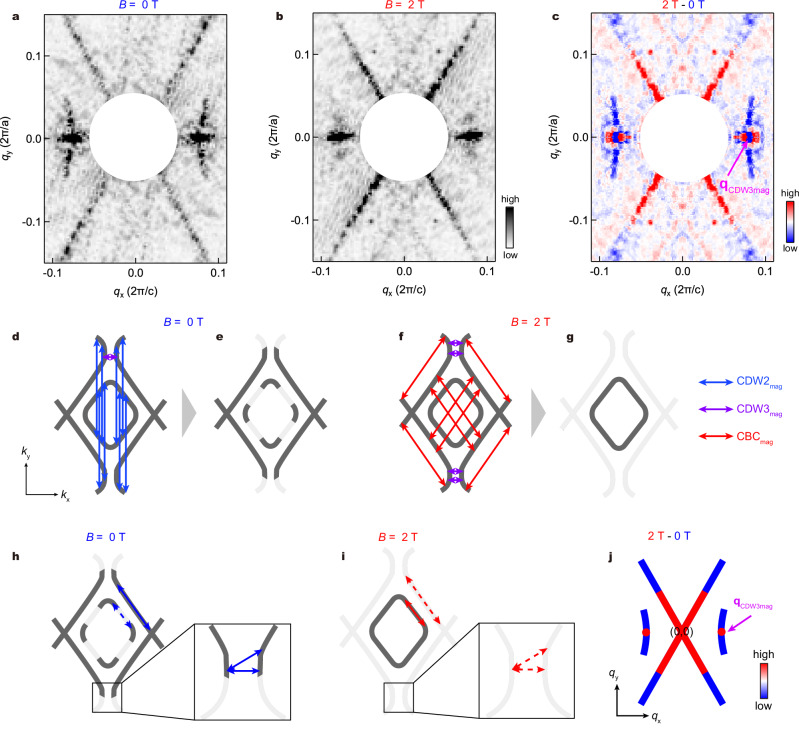
Quasiparticle interference revealing competing Fermi-surface instabilities under an in-plane magnetic field applied along the c axis. Quasi-particle interference (QPI) patterns measured at (*T*, *B*) = (0.3 K, 0 T) (**a**), and (0.3 K, 2 T) (**b**). The set point is −10 mV/1 nA, and lock-in modulation is 3 mV with 961 Hz. Both were symmetrized with respect to *q*_x_ = 0 line, assuming the quasi-tetragonal crystalline symmetry in the non-magnetic phase. Note that the color scale is identical in (**a**) and (**b**). **c** Difference map of the QPI intensity between (0.3 K, 0 T) and (0.3 K, 2 T), showing alternating contrasts. Note that the area around **q** = (0,0) is masked since the major contribution of the irrelevant long wavelength signal is expected to contribute. For transparency, the non-masked data is shown in Supplementary Note 6. Additional QPI features in other momentum regions and their consistency with this model are discussed in Supplementary Note 6. Schematic constant-energy contour evolution owing to the gap opening by the charge orders at *B* = 0 T (**d**, **e**) and *B* = 2 T (**f**, **g**), respectively. The colored arrows indicate the ordering vectors at each phase. **h**, **i** Constrained scattering channels due to the gap opening at *B* = 0 T (**h**) and 2 T (**i**). The solid arrows indicate the possible channels, while the broken arrows indicate the suppressed channels. **j** Expected QPI contrast by subtracting the data at *B* = 2 T by *B* = 0 T, which shows good agreement with the experiments.

To deepen our understanding of the electronic competition in momentum space, we investigate quasiparticle interference (QPI). Motivated by the DOS reconstruction observed at 300 mK around −10 meV across *B*_flop_ (Fig. 4e), we concentrate on comparing high-resolution QPI data taken at 0 and 2 T at the same temperature and energy (Fig. 5a, b). To isolate the field-induced evolution across *B*_flop_, the QPI pattern at 0 T is subtracted from that at 2 T, yielding a differential QPI map (Fig. 5c) that reveals characteristic suppression (blue) and enhancement (red) of QPI intensity with increasing magnetic field. To interpret this red/blue contrast, we consider nesting vectors associated with CDW2_mag_, CDW3_mag_, and CBC_mag_ on approximate constant-energy contours constructed from ARPES measurements of the parent band structure above *T*_N_ (Fig. 1k). At *B* = 0 T, nesting associated with CDW2_mag_ (blue arrows in Fig. 5d) leads to partial gap opening predominantly on the inner pocket (Fig. 5e). In contrast, at *B* = 2 T, nesting associated with CBC_mag_ and CDW3_mag_ collectively produces pronounced gap opening on the outer pocket (Fig. 5f, g).

The essential physics underlying the experimental QPI difference between 0 and 2 T is most clearly manifested near **q** = (0,0). In this region, the QPI is governed by scattering between quasi-one-dimensional, predominantly 5*p*-orbital-derived Fermi surfaces (Fig. 1c, d), whose parallel constant-energy contours generate features that cross the **q** = (0,0) point. Within this framework, the dominant scattering shifts from the outer pocket at *B* = 0 T to a shorter vector within the inner pocket at *B* = 2 T, as indicated by the solid and dashed arrows in Fig. 5h, i. This shift naturally results in a characteristic change in the momentum-space extent of the QPI across **q** = (0,0) (Fig. 5j), which is directly captured in the experimental differential QPI map (Fig. 5c).

The validity of this picture is further supported by its ability to account for the QPI modulation associated with the development of CDW3_mag_ at higher magnetic fields (Fig. 3h). In our model, the corresponding QPI signal is expected to deform and become suppressed as CDW3_mag_ is stabilized, consistent with the *k*-space reconstruction shown in the magnified regions of Fig. 5h, i. The propagation vector **q** = (0, 0.08) associated with CDW3_mag_ is indicated by the arrow in Fig. 5c, around which extended QPI features are observed. While a prominently extended QPI signal is present at *B* = 0 T, this signal is strongly suppressed at *B* = 2 T, concomitant with the enhanced CDW3_mag_ intensity localized at **q** = (0, 0.08) (highlighted regions in Fig. 5a, b). Accordingly, the theoretically expected disappearance (blue-coded) of an arc-like extended QPI feature around **q** = (0, 0.08) at 2 T (Fig. 5j) is faithfully reproduced in the experimental differential QPI map (Fig. 5c). Taken together, the QPI deformations observed between *B* = 0 and 2 T—particularly near **q** = (0,0)—are consistently explained within a minimal modeling framework incorporating CDW2_mag_, CDW3_mag_, and CBC_mag_, providing direct momentum-space evidence for the competition and coexistence of these charge-ordered states.

We next provide a semi-quantitative discussion of the possible relationship between the charge modulation vectors **q**_c_ and magnetic propagation vectors **q**_m_. A natural microscopic connection arises from the Kondo exchange coupling between itinerant Te 5p electrons and localized Ce 4*f* moments^28,29,46,53–55^, where theoretical studies predict that intertwined charge order can emerge from linear combinations of antiferromagnetic propagation vectors **q**_m_^12,15,16^. At zero magnetic field below T_N_, neutron-scattering experiments on CeTe_3_ revealed antiferromagnetic order with propagation vectors **q**_m1_ = (0.17, 0.31) and **q**_m2_ = (0.17, −0.31)^49^. These naturally generate two candidate intertwined charge wavevectors: **q**_m1_ + **q**_m2_ = (0.34, 0) and **q**_m1_ − **q**_m2_ = (0, 0.62). Notably, **q**_m1_ + **q**_m2_ connects more strongly nested regions of the Fermi surface, providing a more efficient route for fermionic energy gain and thereby stabilizing the magnetic charge-ordered state **q**_CDW2mag_ = (0.33, 0) (see blue and purple arrows in Fig. 1k). Although no direct CDW corresponding to **q**_m1_ − **q**_m2_ is observed, combining **q**_m1_ − **q**_m2_ with **q**_CDW1_ = (0, 0.28) yields **q**_CDW3mag_ = (0, 0.08) through the approximate relation **q**_CDW3mag_ ≈ 1 − **q**_CDW1_ − (**q**_m1_ − **q**_m2_). Owing to weaker nesting and the resulting smaller fermionic energy gain, **q**_CDW3mag_ is expected to exhibit a weaker experimental signal at *B* = 0 (Fig. 3h).

Under finite magnetic fields, neutron-scattering information remains limited, and direct determination of the magnetic structure is an important subject for future work. Nevertheless, the emergence of the C_4_-symmetric CBC_mag_ with wavevectors (±0.19, 0.19) (Fig. 3h, right) constrains the possible underlying magnetic configurations. While the C_4_-symmetric magnetically intertwined CDW state has not been reported in magnetically localized systems such as TbTe_3_ and DyTe_3_, in-plane magnetic propagation vectors with |**q**_m_ | ≈ 0.2 r.l.u. are frequently observed in diffraction experiments on these materials^42,43^. This motivates us to speculate that CeTe_3_ may host an analogous but C_4_-symmetric double-**q**_m_ antiferromagnetic state, uniquely stabilized by intertwined Fermi-surface instabilities. One plausible scenario involves double-**q**_m_ antiferromagnetic propagation vectors **q**_m1_ = (0.19, 0) and **q**_m2_ = (0, 0.19). Within this picture, **q**_CBCmag_ = (0.19, ±0.19) can be interpreted as **q**_m1_ ± **q**_m2_, while **q**_CDW3mag_ = (0, 0.08) approximately follows 2**q**_m1_ − **q**_CDW1_ ≈ (0, 0.1). We also note that, when a finite net moment corresponding to (0, 0) component develops, the magnetic and charge propagation vectors coincide (**q**_m_ = **q**_c_), which may contribute to the emergence of CDW3_mag_ and CBC_mag_ above *B*_flop_.

To this point, in addition to conventional **q**_CDW1_ = (0, 0.28), three antiferromagnetic charge-ordered states [with **q**_CDW2mag_ = (0.33, 0), **q**_CDW3mag_ = (0, 0.08), and **q**_CBCmag_ = (0.19, ±0.19)] have been identified in CeTe_3_. Each state is characterized by a distinct nesting vector (Fig. 1k and Fig. 5d, f) and can be described by different linear combinations of the propagation vectors involving **q**_m_ and **q**_CDW1_. From this viewpoint, we regard all three as distinct antiferromagnetic charge-ordered states. Although the precise spin texture of the field-induced state has not yet been directly resolved by neutron diffraction, recent theoretical studies of easy-plane magnets have predicted a wide variety of stable multi-**q**_m_ phases under magnetic fields, including vortex-crystal states^55^.

Although Kondo exchange plays an essential role in mediating the coupling between localized spins and itinerant electrons, our observations point to substantially richer physics beyond this simple picture. The reported Kondo temperature is on the order of 10 K^46^, whereas the magnetic ordering temperature and the spin-flop field observed in this study are only about 1.5 K and 1.5 T, respectively^17^. These energy scales are therefore far too small to account for the pronounced reconstruction of the DOS observed here, which extends over approximately ±30 meV from *E*_F_ across *T*_N_ (Fig. 1g, h) and a comparable ±20 meV across *B*_flop_ (Fig. 4d). This clear separation of energy scales indicates that Kondo exchange alone cannot be the dominant driving interaction. Instead, additional interactions must play a decisive role in shaping the low-energy electronic structure, a feature that should be considered an intriguing aspect of CeTe_3_. Notably, the energy scale of the band deformation identified here (Fig. 4) is comparable to the dispersion kink associated presumably with electron–phonon coupling revealed by Landau-level spectroscopy in the nonmagnetic single-CDW system LaTe_3_^41^. This comparison demonstrates that similarly high-energy electronic coupling is already intrinsic to the Te-based layered electronic structure, even in the absence of 4 *f* moments. In this context, Coulomb interactions between the small electron and hole pockets in this semimetallic system—an electronic configuration closely analogous to that of CeTe_3_ (Fig. 1d and 5f)—are also a candidate driving source of large energy-scale Fermi-surface deformations reminiscent of an excitonic band instability^20,56,57^. Although the present system is not insulating, such effects are expected to be strongly enhanced in van der Waals materials, where reduced dimensionality and weakened electronic screening amplify interaction effects. Taken together, these considerations establish CeTe_3_ as an exceptional two-dimensional platform in which multiple interactions—Kondo exchange, Coulomb correlations, and electron–phonon coupling—cooperatively generate versatile antiferromagnetic charge orders. Through this interplay, tunable and strongly correlated electronic states emerge on a semimetallic Fermi surface, extending well beyond a simple Kondo-driven picture.

From a real-space perspective, this rich landscape offers fertile ground for exploring exotic multi-**q**_m_ magnetic orders. Such textures are widely regarded as a key ingredient for realizing topologically nontrivial magnetic states. They can give rise to large emergent electromagnetic responses, including enhanced transport signals associated with fictitious magnetic fields^10^. Beyond multi-**q**_m_ magnetism, the coexistence of multiple charge orders is also expected to induce unconventional symmetry breaking; for example, nontrivial relative phase shifts between charge orders may generate exotic in-plane inversion symmetry breaking^58^. These possibilities naturally motivate future studies combining microscopic probes with Hall and non-reciprocal transport measurements.

From a complementary momentum-space viewpoint, the realization of versatile multi-**q** antiferromagnetic charge order on a semimetallic Fermi surface opens the door to the exploration of unconventional electronic band structures. In this regime, competing Fermi-surface instabilities may drive the emergence of Dirac-like dispersions, band inversion, and rich momentum-space textures of electronic wave functions. Moreover, the strong spin–charge intertwining uncovered in CeTe_3_ provides a pathway toward unconventional quantum criticality. Whereas in related RTe_3_ compounds such as TbTe_3_ and DyTe_3_ superconductivity emerges near the CDW2 critical point, where magnetism and charge order are largely decoupled^23,35,36^, the magnetically intertwined charge orders identified here point to a qualitatively distinct critical regime and suggest routes toward unconventional superconductivity driven by coupled spin and charge degrees of freedom.

Finally, guided by the general concept of moiré modulation—where the superposition of periodic structures generates distinct wave vectors through linear combinations—our observation of multiple electronic propagation vectors can be viewed, at a conceptual level, as a manifestation of versatile charge–spin intertwined electronic moiré states, rather than a superposition tied to crystalline lattice vectors^59,60^.

In summary, our results establish CeTe_3_ as a versatile platform for realizing multi-**q** antiferromagnetic charge orders, accompanied by large-energy-scale electronic reconstructions characteristic of strongly correlated systems. The observed phenomenology originates from the intertwining of multiple frustration channels—antiferromagnetism, charge ordering, and competing Fermi-surface instabilities—within a semimetallic state. From a conceptual perspective, this study demonstrates that van der Waals antiferromagnetic semimetals offer promising opportunities to explore previously inaccessible, tunable quantum phases in which topology and strong electronic correlations are intrinsically intertwined in two dimensions. From a functional viewpoint, these results provide guiding principles for designing quantum materials that integrate multiple input and output channels, enabling disproportionately large electronic responses to modest external stimuli at the nanoscale in versatile electronic moiré phases.

## Methods

### Sample growth and characterization

Single crystals were grown using a self-flux method. The mixture of the elemental cerium (99.9%) and tellurium (99.999%) chunks in a molar ratio of 1:40 was placed inside the alumina crucible and sealed in an evacuated quartz tube. The ampoule was heated to 900 °C at a rate of 75 °C/hour, held at 900 °C for 24 h, and then slowly cooled to 550 °C at a rate of 2 °C/hour. The crystals were subsequently separated from the remaining flux by centrifugation. Phase purity and stoichiometry are well characterized by XRD and EDS. In the previous report, two characteristic temperatures (*T*_N1_ and *T*_N2_) were pointed out. *T*_N1_ corresponds to short-range magnetic fluctuation, and *T*_N2_ corresponds to long-range antiferromagnetic order. Note that the *T*_N_ in this report corresponds to *T*_N2_ in the literature. We also note that the slightly higher *B*_flop_ inferred from STM/STS at 300 mK compared with magnetometry at 500 mK (Fig. 1i) is naturally attributed to the temperature difference rather than field misalignment (see calibration of magnetic field for Supplementary Note 5).

### STM/STS

#### General experimental conditions

Samples were cleaved in situ under ultra-high vacuum (UHV) conditions at room temperature and transferred directly to the STM head without exposure to air. Based on our experience, cleaving at either 77 K or room temperature does not affect the main conclusions of this study. All samples used in this study were cleaved in situ under UHV at room temperature. Chemically etched W tips were annealed at high temperature in UHV to remove surface oxides. Within the relevant bias voltage range, tip sharpness and stability, as well as density-of-states (DOS) flatness, were carefully calibrated on Au(111) before sample measurements. The non-magnetic nature of the STM tip was verified through dedicated control experiments, ensuring that the observed magnetic-field-dependent *dI/dV* signals originate from intrinsic changes in the sample DOS rather than from spin-dependent tunneling matrix elements. No evidence of tip-condition changes was observed throughout the measurements presented here. Differential conductance spectra were acquired using a standard lock-in technique with a modulation frequency of 961 Hz.

#### Detection of charge order from topography and set-point effect

Acquiring fully energy-resolved STS datasets over the entire magnetic-field range is extremely time-consuming and significantly increases the risk of changes in the tip or surface condition. To ensure reliable data acquisition within a realistic experimental timescale, we therefore use STM topographic imaging as the primary method to track CDW2_mag_, CDW3_mag_, and CBC_mag_. While CDW3_mag_ is best visualized with bias voltages of approximately ±10 meV, the competition between CDW2_mag_ and CBC_mag_ is more clearly captured at ±50 meV. As shown in Fig. 3h, at *B* = 0, the CDW3_mag_-related signal is nearly buried in the background noise at a bias voltage of +50 meV (indicated by the dashed line). With an increasing magnetic field, however, the CDW3_mag_ signal enhancement allows simultaneous visualization of CDW2_mag_, CDW3_mag_, and CBC_mag_ in a single topographic image. Additional experimental details are provided in the Supplementary Note 2.

#### dI/dV spectra

To avoid misleading data interpretation, each *dI/dV* spectrum is spatially averaged over regions of at least 30×30 nm^2^. This averaging procedure also suppresses quasiparticle-interference modulations. Thermal- and magnetic-field-induced extrinsic drift of the probed surface area is carefully corrected, ensuring that spectra acquired under different conditions correspond to essentially the same spatial location.

Strictly speaking, normalization of *dI/dV* spectra involves inherent uncertainty, which limits a fully quantitative analysis of absolute DOS deformation (Fig. 4). Nevertheless, because all spectra are acquired under identical set-point conditions and instrumental energy resolution, they reliably capture relative changes in the DOS near the Fermi level. When comparing spectra acquired at different temperatures, thermal broadening must also be accounted for. To address this qualitatively, we simulate a Gaussian-broadened spectrum corresponding to 4 K based on the 0.3 K, *B* = 0 data. This comparison indicates that thermal broadening plays only a minor role in shaping the main conclusions of this study. Under our experimental conditions, the electronic temperature is sufficiently low to be negligible compared with the sample temperature. Further experimental details are provided in the Supplementary Note 3.

#### Quasiparticle interference (QPI) pattern measurement

Within the scope of this study, we focus on the antiferromagnetic state below *T*_N_ at 300 mK. A systematic temperature-dependent investigation is deferred to future work, where it is expected to be particularly informative. Notably, in the temperature regime above *T*_N_, presumably below ~10 K^46^, we observe indications of an unusual matrix-element effect associated with fluctuating orders^52^. Clarifying its origin requires substantial additional experimental efforts and is therefore beyond the scope of the present study. Further details are provided in Supplementary Note 6.

#### Measurement stability, reproducibility

Measurement stability and reproducibility are verified throughout this study. In particular, before and after acquiring the complete set of magnetic-field- and/or temperature-dependent datasets (Figs. 2–5), we carefully confirm the reproducibility of both tunneling spectra and topographic images, with no discernible changes observed. This demonstrates that the reported results are robust against instrumental drift and measurement history. In addition, the robustness of the main conclusions is further confirmed using more than ten independently prepared STM tips on different sample surfaces (see also Supplementary Note 4).

## Supplementary information

Supplementary Information

Transparent Peer Review file

## Data Availability

The data that support the findings of this study are available from the corresponding author upon request.
