# Supplementary material for: Versatile electronic phases enabled by intertwined multiple frustrations in an antiferromagnetic two-dimensional semimetal
Source: Nat Commun. 2026 Jul 23;17:5981. doi: 10.1038/s41467-026-75048-y (PMC13396192; doi:10.1038/s41467-026-75048-y)
Supplement: Supplementary file 1 — Supplementary Information [file 41467_2026_75048_MOESM1_ESM.pdf]

Supplementary Information for

Versatile electronic phases enabled by intertwined multiple frustrations in an antiferromagnetic two-dimensional semimetal

Y. Fujisawa[1,2*], P. Wu[1,3*], T. Nakamura[1*], R. Okuma[1,4,5], T. Kato[1], B. R. M. Smith[1], D. Ueta[1,6], R. Kobayashi[7], N. Maekawa[7], C-H. Hsu[1], Chandan De[1], N. Tomoda[1], T. Higashihara[1,8], K. Morishita[1,7], K. Sumida[2], K. Miyamoto[2], T. Okuda[2], Z. Y. Wang[3], Y. Okada[1,5]

* equal contribution

[1] *Quantum Materials Science Unit, Okinawa Institute of Science and Technology (OIST), Okinawa 904-0495, Japan.*

[2] *Research Institute for Synchrotron Radiation Science (HiSOR), Hiroshima University, Higashi-Hiroshima 739-0046, Japan.*

[3] *Department of Physics and Chinese Academy of Sciences Key Laboratory of Strongly-coupled Quantum Matter Physics, University of Science and Technology of China, Hefei, Anhui 230026, China.*

[4] *Institute for Solid State Physics (ISSP), The University of Tokyo, Kashiwa, Chiba 277-8581, Japan.*

[5] *JST, PRESTO, 4-1-8 Honcho, Kawaguchi, Saitama, 332-0012, Japan*

[6] *Institute of Materials Structure Science, High Energy Accelerator Research Organization, Tsukuba, Ibaraki 305-0801, Japan.*

[7] *Faculty of Science, University of the Ryukyus, Nishihara, Okinawa 903-0213, Japan.*

[8] *Department of Physics, Graduate School of Science, Osaka University, Toyonaka 560-0043, Japan.*

This supplementary material contains the following.

Supplementary Note 1: Assignment of FFT peaks
Supplementary Note 2: Bias dependence of STM topographs
Supplementary Note 3: Comparison of dI/dV spectra under varying temperature and magnetic field
Supplementary Note 4: Measurement stability and reproducibility
Supplementary Note 5: Calibration of the in-plane magnetic-field direction
Supplementary Note 6: Extended QPI data

Supplementary Note 1: Assignment of FFT peaks

Major peaks

The Fourier transforms (FTs) of STM topographs and dI/dV maps contain multiple Bragg and superlattice peaks, and the main conclusions of this study are based on a qualitative but systematic identification of all resolvable peaks (Figs. S1–S3). Peaks are considered genuine only when they appear assuming quasi-tetragonal crystal symmetry. At (T, B) = (4 K, 0 T) and (0.3 K, 0 T), the dominant FFT peaks can be consistently indexed as linear combinations of the primary modulation vectors $\mathbf{q}_{CDW1}$ = (0, 0.28), $\mathbf{q}_{CDW2}$ = (0.33, 0), and $\mathbf{q}_{CDW3}$ = (0, 0.08), together with the Bragg vectors (n, m), where n and m are integers. At (T, B) = (0.3 K, 0 T), most peaks can alternatively be indexed using linear combinations of $\mathbf{q}_{CDW1}$, $\mathbf{q}_{CDW3}$, $\mathbf{q}_{CBC1}$ = (0.19, 0.19), and $\mathbf{q}_{CBC2}$ = (0.19, −0.19).

Minor peaks on the q_y=1/2 line, under a finite magnetic field

At this point, unindexed peaks remain on the q_y=0.31 and 0.5 lines under a finite magnetic field (Fig. S3a). This component cannot be generated from linear combinations of the established charge order and 1 x 1 lattice modulation vectors, indicating that it represents an independent ordering tendency that emerges only under a magnetic field.

We find that an additional vector q* ~ (1/2, 0) can account for these weak peaks at B = 2 T and 0.3 K (Fig. S3). The corresponding **q*** vector is close to the commensurate (1/2, 0) magnetic component reported in neutron studies of other RTe_3 compounds such as $TbTe_3$ and $GdTe_3$ [43,45], where the order is predominantly localized magnetic character and produces minimal renormalization of the itinerant electronic structure (Fig. 1h). This comparison suggests that **q*** is likely associated with a field-stabilized order of localized Ce 4f electrons with a finite net moment. While both the propagation vector corresponding to $CDW3_{mag}$ ($\mathbf{q}_{CDW3mag}$) and **q*** + $\mathbf{q}_{CDW3mag}$ are plausible from the viewpoint of Fermi-surface nesting, as they involve nearly identical portions of the Fermi surface, the substantially weaker intensity of **q*** compared with $\mathbf{q}_{CDW3mag}$ motivates us not to emphasize **q*** in the main text.

Supplementary Note 2: Bias dependence of STM topographs

To validate the use of STM topography for identifying charge order (Figs. 2 and 3), we examine the bias dependence of STM images. Topographs acquired at various set-point biases (Fig. S4a) are analyzed, and the Fourier intensity at $\mathbf{q}_{CDW3mag}$ and $\mathbf{q}_{CBCmag}$ is plotted as a function of the bias voltage (Figs. S4c,d). We also show the FFT intensity at $\mathbf{q}_{CDW2mag}$, $\mathbf{q}_{CDW3mag}$, and $\mathbf{q}_{CBCmag}$ as a function of spectroscopic bias taken from the *dI/dV* mapping as shown in Fig.S4b,c,d, respectively.

As shown in Fig. S4c, the $CDW3_{mag}$ modulation is most clearly visualized in STM topographs and dI/dV mappings at relatively low bias voltages within the explored range. On the other hand, the $CDW2_{mag}$ and CBC_{mag} components exhibit stronger contrast at higher bias voltages. This trend is broadly consistent with the spectroscopic weight of these modulations being observed away from the Fermi level (Fig. 4),

suggesting that STM topographs at higher bias voltages capture their spatial modulation more effectively. Thus, we choose a set-point bias of V_{set} = −10 mV when we need to track the intensities of $CDW2_{mag}$ and $CDW3_{mag}$ efficiently, since both modulations remain visible in the FFT of the topographs at this bias (Fig. 2j). For measurements aimed at highlighting the competition between $CDW2_{mag}$ and CBC_{mag}, we adopt a higher set-point bias of +50 mV, which maximizes their spatial contrast (Fig. 3h). While the apparent FFT intensity variation exists in Fig. S4d due to the set-point effects, the presence or absence of each modulation wave vector is robust against the specific choice of bias. As long as the datasets used to extract essential physical information are obtained under unified setpoint conditions, reliable trends in the stabilization and destabilization of the antiferromagnetic charge orders can be established. Consequently, each dataset provides a self-consistent and reliable basis for the main conclusions of this manuscript.

Figures S5 show the FFT intensity evolution with and without normalization by the Bragg peak intensity. Figure S5a presents the data normalized by the Bragg peak intensity, while Figure S5b shows the corresponding data without normalization. As demonstrated in Fig. S5b, even without normalization, the same major trends observed in the normalized data (Fig. 3h) are clearly visible. The primary motivation for applying normalization is to minimize tip-related effects, which can introduce extrinsic anisotropic intensity in STM images. Accordingly, as in Fig. 3h, each CDW-related signal is normalized by the Bragg peak intensity along the corresponding crystallographic direction.

Supplementary Note 3: Comparison of dI/dV spectra under varying temperature and magnetic field

Spatial averaging

To achieve reliable *dI/dV* shape comparison (as in Fig. 1g,l and Fig. 4), to minimize the impact of spatial inhomogeneity, spectra are spatially averaged over areas larger than 30 nm using a dense grid of approximately 30 × 30 points. This averaging suppresses quasiparticle interference, defect-related variations, and charge-order modulations. Although a small conductance variation accompanies CDW formation, its magnitude and characteristic length scale are negligible compared with the averaging area (Fig. S6). Based on these curves, we compare the reliable *dI/dV* shape. Notably, if we observe any change in tip condition during a single data set, we restart the experiments to investigate the *dI/dV* spectral evolution.

Temperature broadening

To distinguish intrinsic electronic reconstruction from thermal broadening across $T_N \approx 1.5$ K, the 0.3 K spectrum is thermally broadened using a Gaussian corresponding to 4 K and compared with the measured 4 K spectrum. As shown in Fig. S7, the experimentally observed spectral changes are substantially larger than those expected from thermal broadening alone, demonstrating that the reconstruction is intrinsically linked to the antiferromagnetic transition.

Supplementary Note 4: Data reliability

Measurement stability and reproducibility are verified throughout this study. Tunneling spectra and topographic images acquired before and after the complete set of temperature- and magnetic-field-dependent measurements (Figs. 2–5) show no discernible changes, confirming robustness against instrumental drift and measurement history. The main conclusions are further validated using more than ten independently prepared STM tips on different sample surfaces. Figure S8 shows measurements acquired under identical experimental conditions within the same field of view, where a characteristic impurity remains unchanged, confirming both tip stability and positional reproducibility. Distinct charge orders (CDW1, $CDW2_{mag}$, $CDW3_{mag,}$ and CBC_{mag}) are reproducibly observed in both topographic and *dI/dV* maps, with corresponding Fourier transforms confirming their robustness. Figure S9 further demonstrates that the topographs obtained before and after the magnetic-field-dependent measurements are nearly identical. Also, the FFT intensities before and after the measurement are shown in Fig. S9e.

Supplementary Note 5: Calibration of the in-plane magnetic-field direction

The in-plane magnetic field orientation is calibrated using $NbSe_2$ before measurements on $CeTe_3$. In our setup, a tilted magnetic field produces elongated vortex cores in $NbSe_2$, whose orientation directly reflects the in-plane component of the applied field. Zero-bias conductance maps acquired under different field orientations (Fig. S10) show that the vortex tails align with the applied in-plane direction in all cases, confirming the accuracy and reliability of the magnetic-field alignment used in this study.

Supplementary Note 6: Extended QPI data

To support the proposed Fermi surface evolution, the extended QPI data in wider q-space are presented here. The data shown in Fig. 5 is acquired within a 200 x 200 nm^2 field of view to investigate at high q resolution. The STM topograph and corresponding conductance map, and its Fourier transformed image taken at 0.3 0 T and 2 T are shown in Fig. S11a-f, respectively. As seen in the magnified images, no detectable change in the tip condition was observed. While we focused on the (0,0) region, marked by a black rectangle in the main text (Fig. 5), the region around (0,1), highlighted by the red rectangle, also provided a consistent picture (Fig. S11g-l). Figures S11g and S11h show magnified FT images obtained at B = 0 T and 2 T, respectively. The intensity variation is visible along the dashed black line.

To highlight these differences, we compute the differential QPI map shown in Fig. S11i, with representative scattering channels indicated for discussion below. These scattering channels are assigned phenomenologically, as illustrated in Fig. S11j. The observed discontinuity in QPI intensity between $\mathbf{q}_2$ and $\mathbf{q}_3$ is consistent with the boundary separating inter-pocket and intra-pocket scattering processes. Experimentally, the suppression of $\mathbf{q}_1$ at B = 0 T is consistent with gap opening on the inner hole pocket, while the other channels remain relatively intact (Fig. S11k). In contrast, at 2 T, the $\mathbf{q}_2$, and $\mathbf{q}_3$ signals are relatively suppressed, whereas the $\mathbf{q}_1$ signal becomes dominant. Thus, observations in the $\mathbf{q} \approx (0, 1)$ region are also consistent with the Fermiology proposed in the main text, which is centered around $\mathbf{q} \approx (0, 0)$.

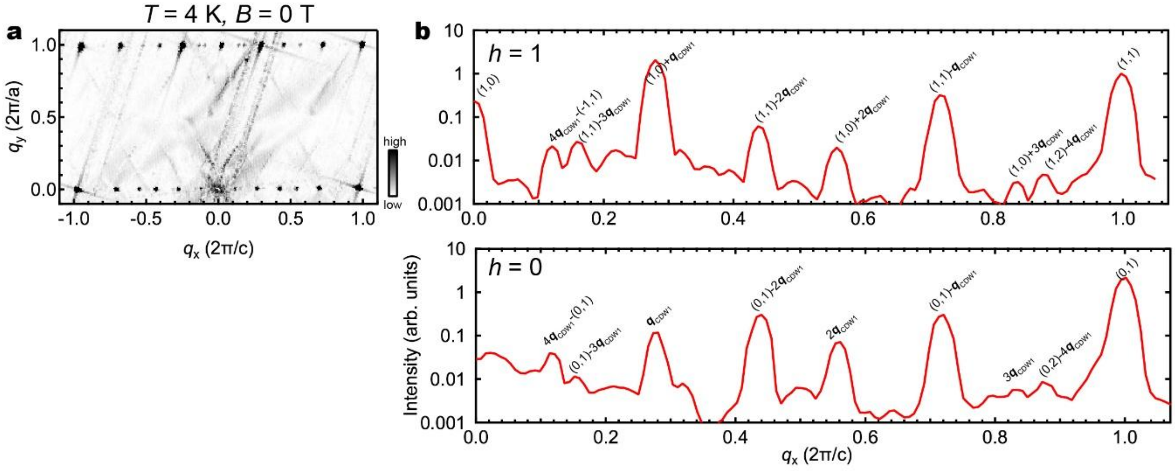

Fig. S1

Assignment of FFT peaks at 4 K.

a Fourier transformed image of the STM image taken at 4 K. **b** Line profiles of a for specific *h* lines (see the *h* values indicated inside each figure). The setpoint is +50 mV/4 nA.

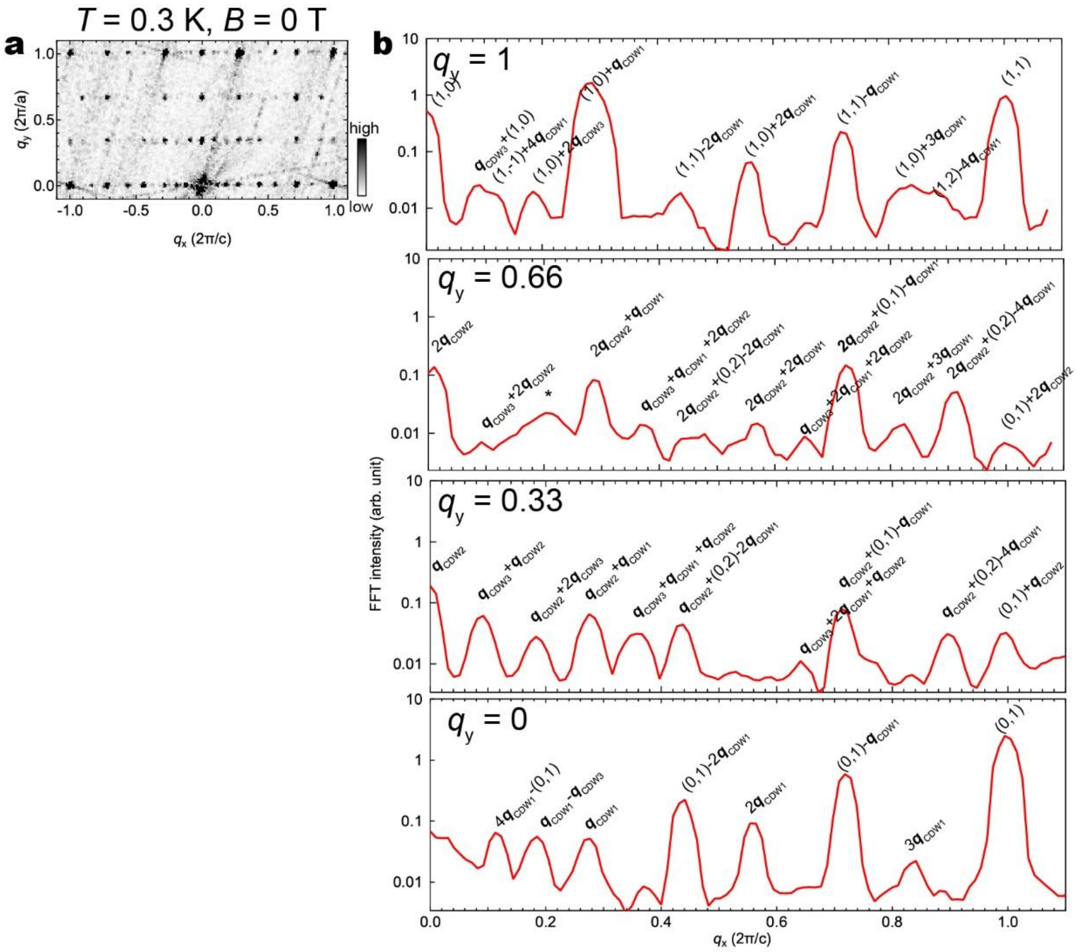

Fig. S2

Assignment of FFT peaks at 0.3 K and 0 T.

a Fourier transformed image of the STM image taken at 0.3 K and 0 T. **b** Line profiles of a for specific *h* lines (see the *h* values indicated inside each figure). The setpoint is +50 mV/5 nA. Note that asterisks (*) denote peaks arising from either scan noise or quasiparticle interference, both of which are unrelated to the present study.

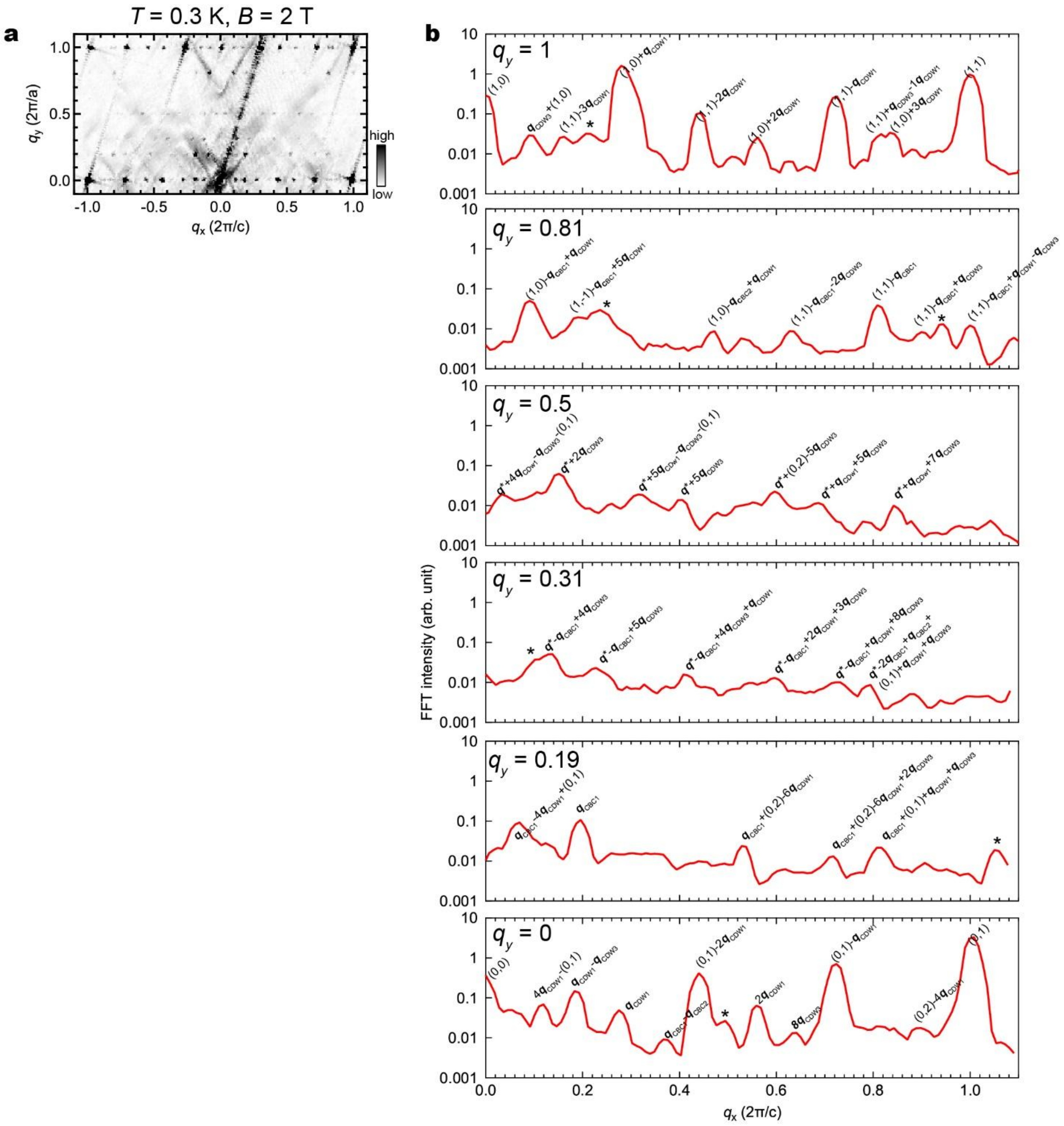

Fig. S3

Assignment of FFT peaks at 0.3 K and 2 T.

a Fourier transformed image of the STM image taken at 0.3 K and 2 T. **b** Line profiles of a for specific *h* lines (see the *h* values indicated inside each figure). The setpoint is +50 mV/2.5 nA. Note that asterisks (*) denote peaks arising from either scan noise or quasiparticle interference, both of which are unrelated to the present study.

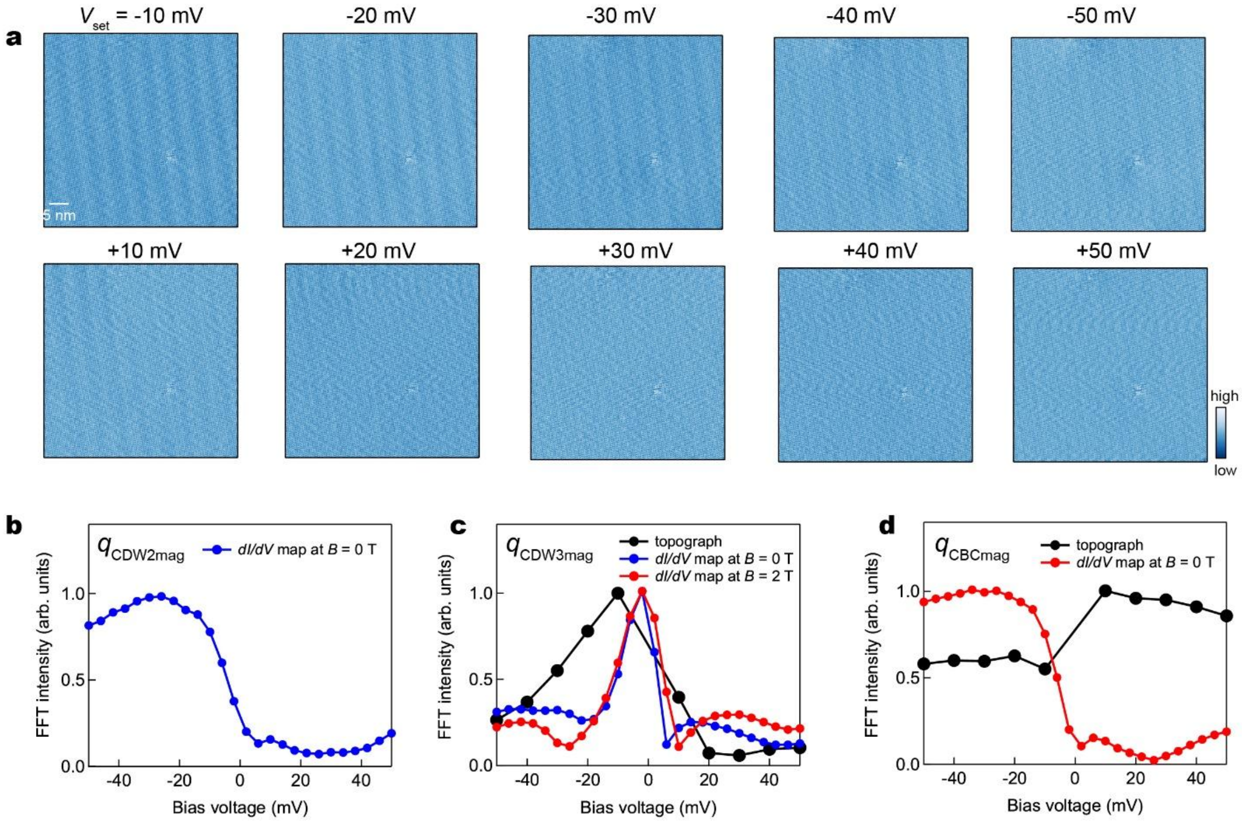

Fig. S4

Bias dependence of STM topography and FFT intensity.

a, STM topographs acquired at various set bias voltages (V_{set}) while keeping the tunneling current fixed at 1 nA.

b–d, FFT intensity plotted as a function of bias voltage normalized by the maximum values. For the data obtained from STM topographs, the voltage corresponds to the set bias voltage used during image acquisition. For data derived from dI/dV maps, the voltage corresponds to the spectroscopic bias used in the differential conductance measurements under fixed setup conditions. The STS data are taken from Figs. 2 and 3.

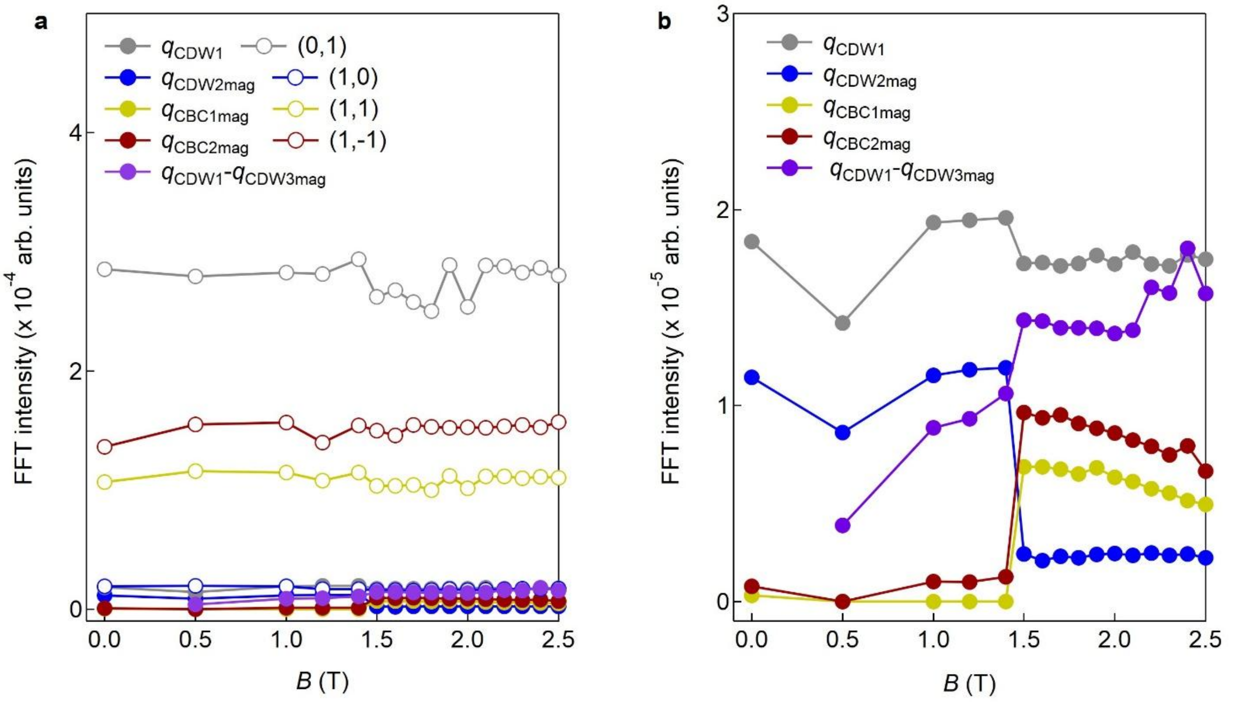

Fig. S5

Magnetic-field dependence of Fourier peak intensities.(a) Magnetic-field dependence of the Fourier peak intensities corresponding to the charge-order wave vectors and Bragg peaks.

(b) Magnified view of panel (a), showing the Fourier peaks with intensities below 3×10^{-5}arbitrary units.

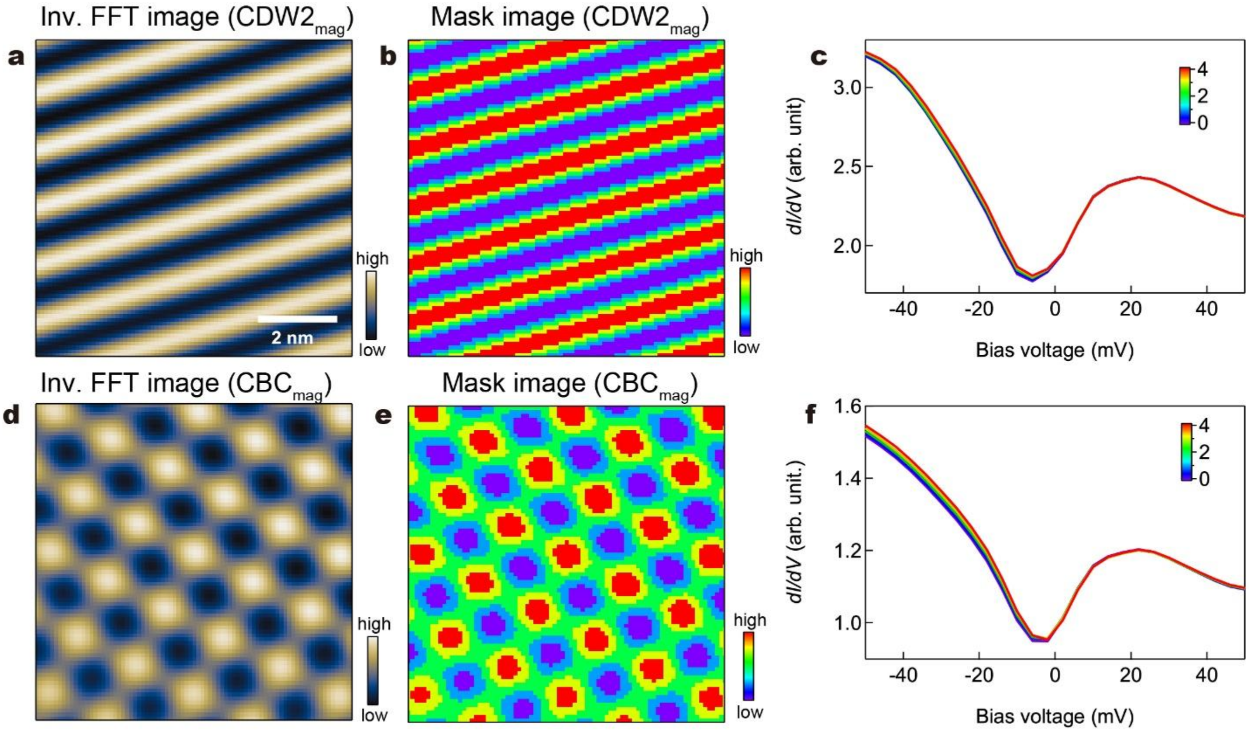

Fig. S6.

Spatial dependence of the dI/dV spectra associated with the CDW2$_{mag}$ and CBC$_{mag}$ phases.

(a,d) Fourier-filtered real-space *dI/dV* maps at -18 mV obtained by inverse Fourier transformation using the characteristic wave vectors $\mathbf{q}_{CDW2}$ = (0.33, 0) and $\mathbf{q}_{CBC}$ = (±0.19, 0.19), respectively.

(b,e) The filtered maps are divided into five representative regions based on modulation amplitude, indicated by different colors.

(c,f) Spatially averaged *dI/dV* spectra obtained by averaging spectra within each region shown in (b,e).

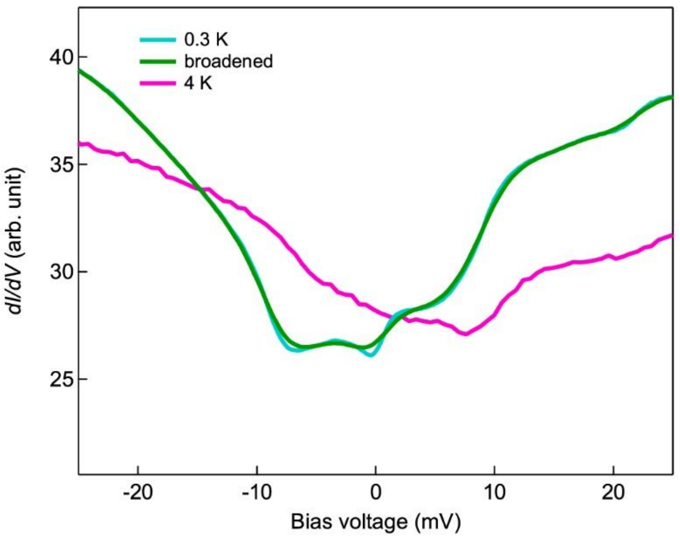

Fig. S7.

Consideration of the thermal broadening effect on the tunneling spectrum.

To obtain the spectrum including the thermal broadening effect (green curve), the spectrum measured at 0.3 K (cyan curve) is convolved with a Gaussian function whose full width at half maximum (FWHM) is given by $3k_B\sqrt{4.2^2 - 0.3^2} \approx 1.1\, meV$.

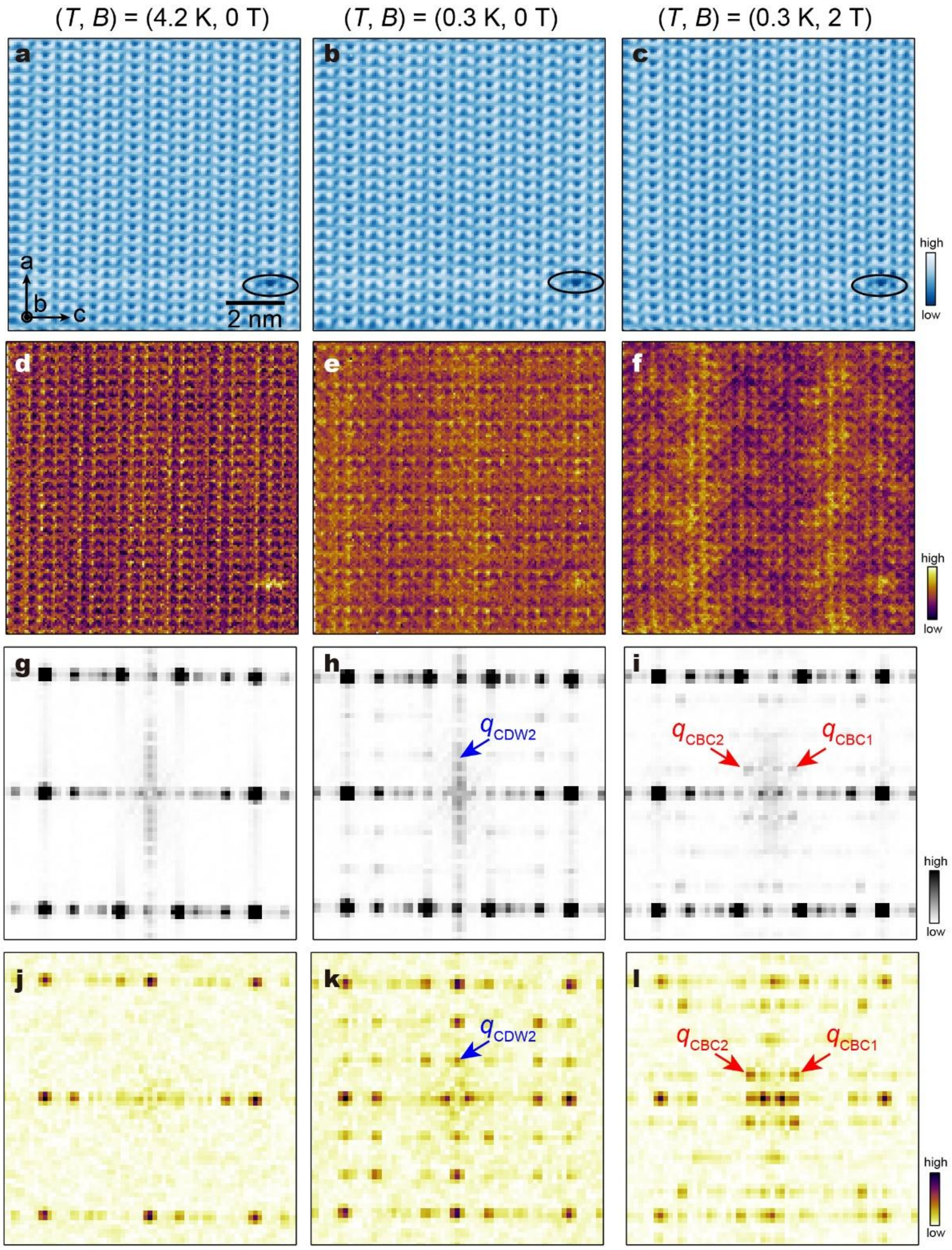

Fig. S8

A single dataset taken with fixed experimental conditions.

a-c, STM topographs taken at various temperatures and magnetic fields, while keeping the same set-up conditions, same field-of-view, and the same tip condition. The set point is -60 mV/200 pA.

d-f Simultaneously obtained dI/dV conductance maps at +18 mV. The lock-in amplitude is 3 mV at 961 Hz.

g-l, Fourier transform images of a-f, respectively.

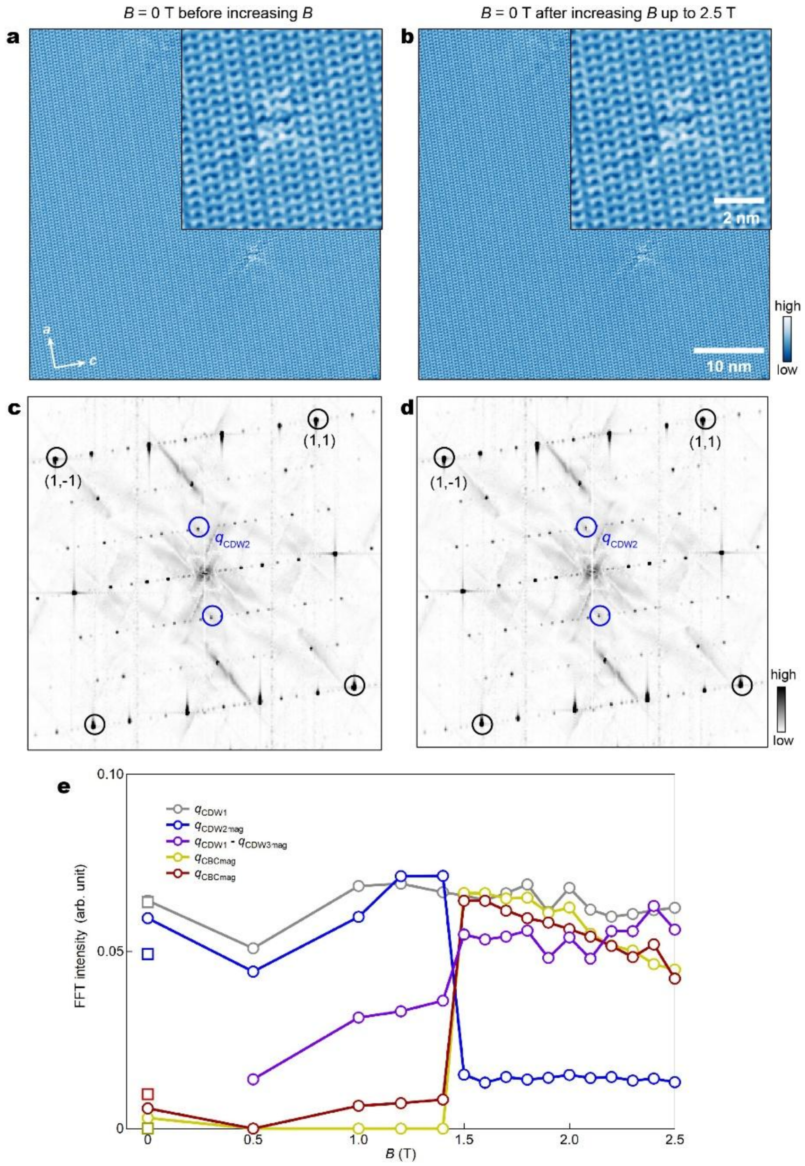

Fig. S9

Data reproducibility across B-evolution experiments.

a,b, STM topographs at *B* = 0T taken before and after ramping the magnetic field up to 2.5 T. The set point is +50 mV/2.5 pA.

c,d, Fourier transform images of a and b, respectively.

e, Magnetic field evolution of the FT peak intensities at $\mathbf{q}_{\mathrm{CDW1}}$, $\mathbf{q}_{\mathrm{CDW2mag}}$, $\mathbf{q}_{\mathrm{CDW3mag}}$, and $\mathbf{q}_{\mathrm{CBCmag}}$, which are normalized by the Bragg peaks at (q_x,q_y) = (1,0), (0,1), (1,0), and (±1,1), respectively, extracted from topographic images. The circle symbols are from the STM topograph after ramping the magnetic field up to 2.5 T. The intensity reproduces the data reasonably well before increasing the magnetic field, suggesting robust tip stability.

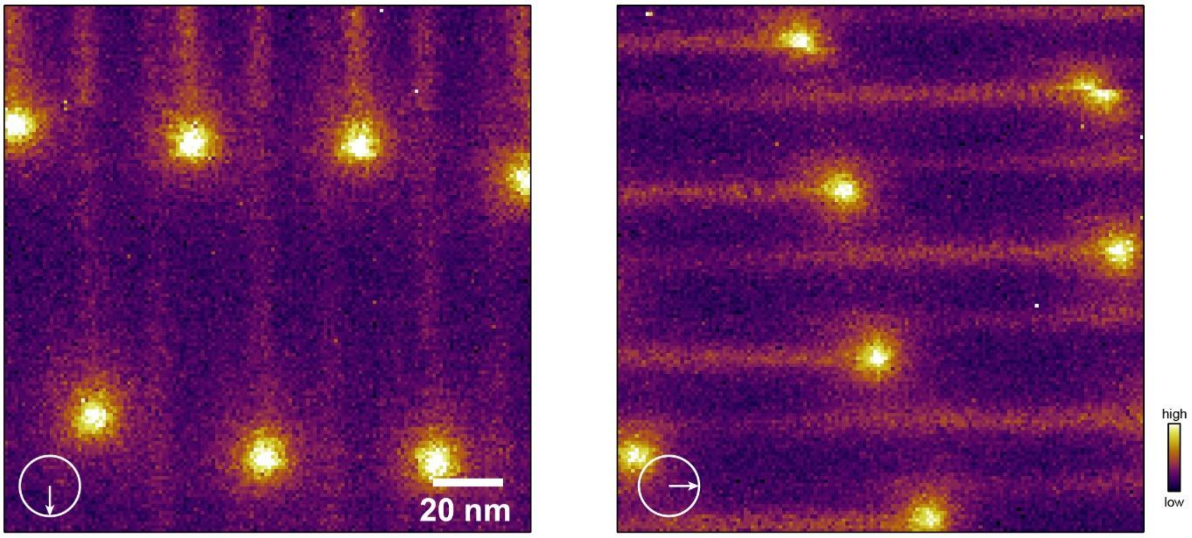

Fig. S10

The in-plane field orientation calibration using $NbSe_2$.

Zero-bias conductance maps acquired with a setpoint of +20 mV / 100 pA and a lock-in amplitude of 0.5 mV. The magnetic field magnitude was fixed at 0.2 T and tilted by 80 degrees from the surface normal. The white arrows indicate the direction of the in-plane field component.

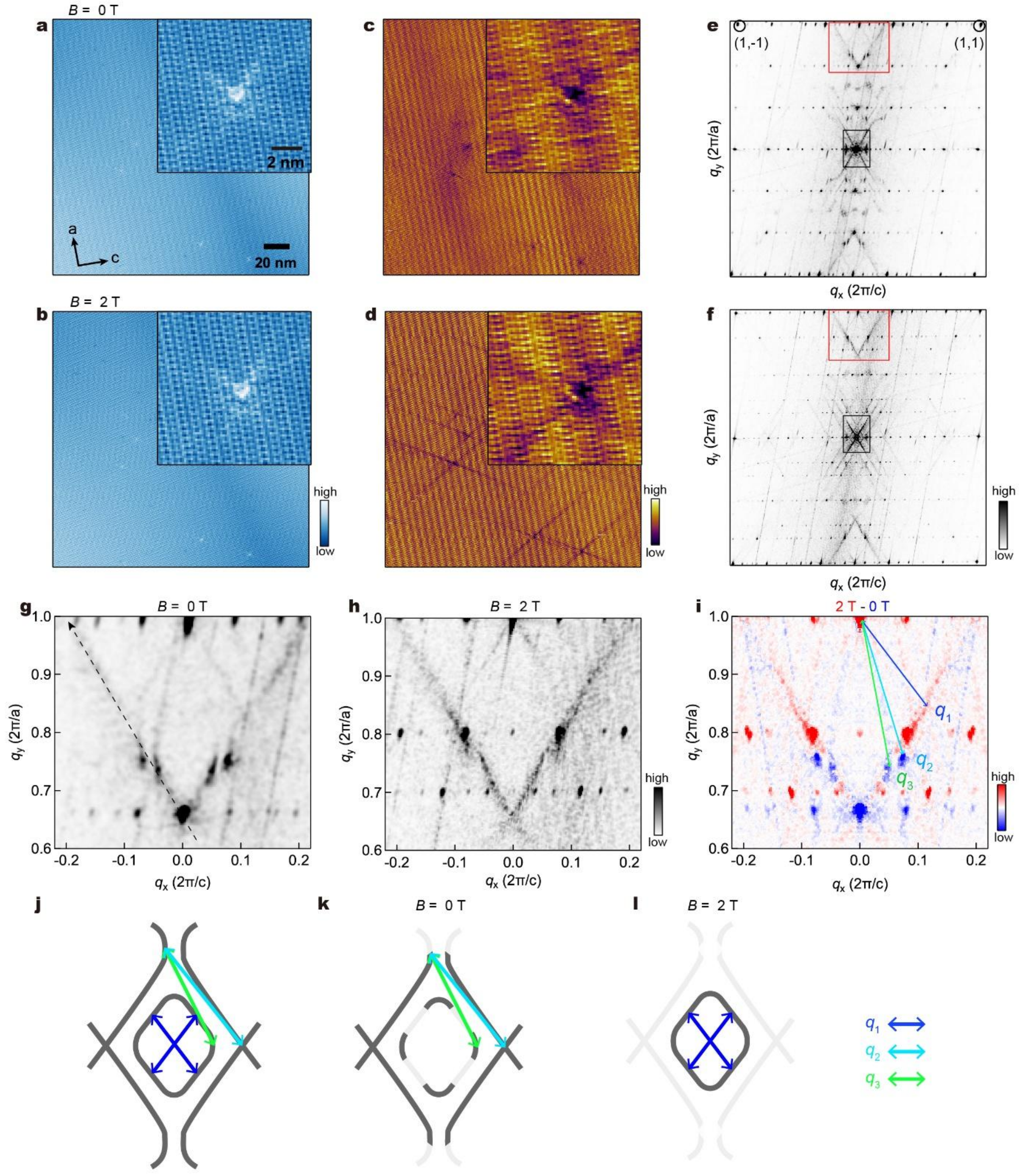

Fig. S11

Extended QPI around q ≈ (0,0) and its magnetic-field evolution

(a,b). Large-area STM topograph (200 × 200 nm^2) acquired at 0.3 K and 0 T (a), and 2 T (b), respectively. The setpoint is -10 mV/1 nA. The inset images are magnified views of the main panel, confirming the stability of the STM tip during the experiments.

(c,d) Simultaneously obtained differential conductance (*dI/dV*) maps at -10 mV.

(e,f) Fourier-transformed conductance maps. The (0,0) region indicated by the black box is discussed in the main text, while the red dashed box marks the **q** ≈ (0,0) region analyzed here.

(g,h) Zoomed-in FT-QPI maps around **q** ≈ (1,0) measured at 0 T (g) and 2 T (h), respectively.
(i) Differential QPI map (2 T − 0 T) highlighting the magnetic-field-induced redistribution of spectral weight. The data is symmetrized with respect to *the* q_x=0 line*,* assuming the quasi-tetragonal symmetry.
(j,k,l) Schematic Fermi surface deduced from the ARPES image (j), with partial gap opening expected from the ordering vectors at B = 0 T (k) and B = 2 T(l), respectively.
