# Supplementary material for: Versatile electronic phases enabled by intertwined multiple frustrations in an antiferromagnetic two-dimensional semimetal
Source: Nat Commun. 2026 Jul 23;17:5981. doi: 10.1038/s41467-026-75048-y (PMC13396192; doi:10.1038/s41467-026-75048-y)
Supplement: Supplementary file 2 — Transparent Peer Review file [file 41467_2026_75048_MOESM2_ESM.pdf]

nature portfolio

Peer Review File

Versatile electronic phases enabled by intertwined multiple frustrations in an antiferromagnetic two-dimensional semimetal

Corresponding Author: Professor Yoshinori Okada

This file contains all reviewer reports in order by version, followed by all author rebuttals in order by version.

Version 0:

Reviewer comments:

Reviewer #1

(Remarks to the Author)
The manuscript Versatile multi-q antiferromagnetic charge order in correlated vdW metals by Fujisawa, et al. presents STM data on the layered antiferromagnet $CeTe_3$. The main findings of this work are
1) Appearance of the stripe charge order across magnetic ordering temperature (Neel temperature)
2) Appearance of the checkboard charge order and suppression of the stripe charge order across spin flop transition.

Discovery of charge orders intertwined with magnetic order is one of the exotic phenomena in condensed matter physics. Also, the manuscript is written clearly and is well-organized. In principle I am in favor of recommending this paper for publication if authors successfully address my concerns and comments below.

Major comments
1) Further insights on underlying mechanism of intertwining: It would be much more insightful and impactful if the authors characterize microscopic structure of the underlying magnetic ordering below Neel temperature and above spin-flop magnetic field (and propose underlying mechanism of intertwined charge and magnetic order + competition between CDW2_mag and CBC_1,2). In this regard, would it be possible for authors to conduct spin-polarized STM as in study of other RTe3 system [npj Quantum Mater. 9, 47 (2024)]? I understand it could be a long journey so If the authors have no previous experience of spin-polarized STM, this comment can be skipped over.

2) Novelty: Several recent publications showcase the intertwining of magnetic and electronic orders in RTe3 materials. One example is from X-ray and neutron studies of $TbTe_3$, published in Chillal, et al., Phys. Rev. B 102, 241110(R) (2020). Another example includes $CeTe_3$ itself, as seen in a magnetotransport study published in Higashihara, et al., Phys. Rev. B 109, 134404 (2024). In the paper by Higashihara, et al., the resistivity and carrier concentration in $CeTe_3$ are shown to change abruptly below the magnetic transition, while remaining relatively steady in $TbTe_3$. Although STM visualization of changes in density-of-states maps demonstrated in the present work does provide further information, could the authors show more directly how the present work yields novel insights justifying publication in Nat. Commun.?

3) Data repeatability: Having looked at both the main text and the supplementary material, the presented data appears to have come from a single set of measurements. The sample was measured at 4 K, it was then cooled down to 300 mK, and then an in-plane magnetic field up to 2.5 T was ramped up in increments while the sample was held at 300 mK. For a journal of Nat. Commun.'s caliber showing repeatability would make the arguments a lot more convincing. For example, did the Fourier intensities match when the field was ramped back down to 0 T while at 300 mK? It has been shown, for example, in Fu, Kraft, et al., Phys. Rev. B 94, 205101 (2016), that STM measurements of Fourier intensities in RTe3 materials can be highly sensitive to tip conditions. Given this, data addressing repeatability would make the arguments much more watertight.

Minor comments
1) All the dI/dV spectra shown are between -30 mV and +30 mV or so. Can the authors show 1-2 larger energy range spectra as well? It would be great to include dI/dV comparison for a wider range of energy to show from which energy the DOS across T_N starts to converge.
2) Missing setpoint information in Fig.3e

3) Regarding spin-flop phase, can author present multiple energy slices of the dI/dV map so that one can check at which energy the checkerboard pattern of CBC1,2 is most prominent? Also, can the author shows dI/dV map at a fixed energy (maybe at -6 meV as the CDW2_mag was most clearly visualized?) with the magnetic field below and above spin-flop transition, so that one can visually see the competition of CDW2_mag and CBC in real space.
4) It is stated in the manuscript that all dI/dV spectra are normalized by the area between 0 mV and -30 mV. It would be useful to also see a comparison with spectra which are not normalized using this procedure.

In summary (for the editor), I recommend publishing this work if the authors address my comments above.

Reviewer #2

(Remarks to the Author)
By using scanning tunneling microscopy and spectroscopy at 300 mK, the authors uncover two competing states in vdW metal $CeTe_3$: charge-ordered in-plane antiferromagnetic phases forming stripe and checkerboard patterns. Remarkably, the competition between them is tuned through a modest in-plane magnetic field (~1.5 T), revealing significant cooperative phenomena between frustrated antiferromagnetism, charge order, and competing Fermi surface nesting. While the results are interesting, however, some important issues need to be addressed before considering the publication in nature communications.
1. Figure 1b shows for CeTe3, no CDW2 phase exists; and TN is about 1 K, which is consistence with Fig.1h. However, in Fig.2, the author observed CDW2mag at 300 mK. Does it mean the refence 26 is wrong, or just because not going lower temperature? Is CDW2mag is same as CDW2? The authors state “Although its nondispersive wavevector (0.33, 0) resembles CDW2 found in the other RTe_3 compounds, its exclusive onset below TN points to a different mechanism, justifying the term CDW2mag due to its connection to magnetism.” The onset below TN cannot justify the CDW2mag due to magnetism, it can be other mechanism, for example the evolution of phonon spectrum. The author needs more evidence to clarify the magnetic origination of CDW2.
2. Please note what the blue arrows in Fig. 2e stand for. And what is the abbreviation of “r.l.u.”? and also please mark the pathway of line-cut profile of the measurement of Fig. 2f in Fig. 2d. If assuming the (0.0) position is for the central spot, what are the rest of three spots in panel d? And the label of horizontal axis in 2f is missing.
3. Fig. 3g shows the energy dependence of the peak intensities associated with CDW2mag relative to the Bragg peak. However, it doesn’t consistence with Fig. 2f, I don’t see the intensity peak at +15 meV in f panel. please explain.
4. Is there any particular reason to compare the line-cut profile of the Fourier signal at ±22 meV in Fig. 2h?
5. Currently, FT image in Fig. 3b is taken from topographic image of a panel. why not taken from dI/dV mapping of d panel, which at least could remove topographic contribution. Actually, all topographic images are look very similar. The lattice of surface Te contribute to the image mostly, while the electronic DOS contribute a little.
6. A critical concern is, all topographic images of Fig. 1g, Fig. 3a, the Fig. 2a and 2c, the measuring setpoints are different. Thus the distance of tip/sample are different, may cause some tip effect on the imaging. The authors need exclude such effect.
7. How is the spatial evolution of dI/dV spectra? When the author states the Fig. 4a shows evolution of the spatially averaged dI/dV spectra under in-plane magnetic fields, please clarify how the measurement are performed and how spatially average being done.
8. Most importantly, the author claims the observed CDW2mag and CBCmag are due to magnetic orders, however, there is no direct evidence to support it. What the author have is the B dependence of ICDW2/IBragg and ICBC1,2/IBragg. It is better to use spin-polarized STM technique to resolve the related spin texture.
9. Magnetic field of 1.5 T induce a spin-flop, the author assume that a net ferromagnetic component emerges from the AF background at magnetic fields above Bflop. While the author attributes the competing nature of CDW2mag and CBCmag states to small-perturbation driven wide energy range deformations of DOS around EF. As we know, Bflop (~1.5 T) and TN (~1.5 K) is a relatively small energy, I doubt it can drive a FS deformation. The author gives many possible mechanisms, such as via Zeeman-induced band splitting, a spin-orbital effect or electron-phonon coupling. The author need provide a confirmative speculation with solid evidence.
Overall, I think the experimental results is not enough to support their claim. Therefore，I suggest the author need provide more solid experimental evidence to claim the observation of multi-q antiferromagnetic charge order in CeTe3.

Reviewer #3

(Remarks to the Author)
I co-reviewed this manuscript with one of the reviewers who provided the listed reports. This is part of the Nature Communications initiative to facilitate training in peer review and to provide appropriate recognition for Early Career Researchers who co-review manuscripts.

Reviewer #4

(Remarks to the Author)
The authors present STM/STS measurements on the cleaved surface of the van der Waals compound CeTe3, conducted over a temperature range from 4 K to 300 mK and under in-plane magnetic fields up to 2 T. Fourier transforms of both the topography and the dI/dV maps reveal a CDW with wave vector q = (0, 0.28) in the normal state at 4 K. At lower temperatures, below the antiferromagnetic transition, additional periodicities appear: q = (0.33, 0) at zero field and q = (0.19, +-0.19) under in-plane fields exceeding the spin-flop transition. These additional charge orders are discussed in

relation to nesting vectors on a proposed phenomenological Fermi surface. The manuscript also includes STS data across the AF phase, both above and below the spin-flop transition.

The experimental results and the interpretation are generally reasonable. However, the absence of quasiparticle interference measurements or electronic band calculations is a significant omission. Without these, it is difficult to convincingly disentangle the interplay between charge and magnetic orders in $CeTe_3$. Additionally, the manuscript lacks a strong connection to the more exotic or topological multi-q phases referenced in the abstract and introduction. For these reasons, I believe the work may be more appropriate for a specialized journal rather than Nature Communications.

Comments:
1. Prior studies by the authors (e.g., Fig. 4 in Ref. 11) report two AF transitions at approximately 3 K and 1 K. Does the sample in this study not exhibit a transition at 3 K? The lack of experimental data near 3 K and the absence of discussion regarding this known feature raise concerns about completeness and scientific transparency.

2. The authors state that they normalize I_{CDW} by I_{Bragg}, but the rationale for this is unclear. Wouldn't it be more appropriate to analyze the normalized conductance $L=(dI/dV)/(I/V)$, in order to mitigate the set-point effect? Clarification is needed.

3. What is the orientation of the applied in-plane magnetic field with respect to the crystal axes? The discrepancy in the spin-flop field between magnetization and STM measurements suggests that the field may not lie strictly along the crystallographic axes (e.g., a deviation from the c-axis in the ac plane). This ambiguity makes it difficult to interpret the STM results in the context of the underlying magnetism. It is important to experimentally rule out the possibility that the observed features arise from the misalignment of the magnetic field.

4. The set-point parameters for the 4 K and 0.3 K data in Fig. 1f appear inconsistent. These should be aligned ideally.

5. It is unclear which "l" the line cut in Fig.2f is at.

Version 2:

Reviewer comments:

Reviewer #1

(Remarks to the Author)
The authors have made a number of significant improvements in the revised manuscript, addressing a number of the points raised after the first review round.

Specifically, the original manuscript had no quasiparticle interference (QPI) data, while the revised one has QPI measurements showing Fermi surface instabilities under the in-plane magnetic field. Moreover, the revised manuscript has shown more data to ensure repeatability, as with the measurements after ramping down the field to 0 T from 2.5 T presented in Fig. S9.

I am in favor of recommending this manuscript for publication if the remaining minor points are addressed:

1. The response file notes that raw dI/dV data which are not normalized by the area between 0 mV and -30 mV (as in the original manuscript), are shown in Fig. S7b. However, there is no Figure S7b. Can this be clarified? Are all the spectra shown in the manuscript now raw, or is a normalization being applied?

2. It isn't clear from the text why the new title highlighting 'electronic moiré phases' accurately describes the impact of this work. I cannot find a description of what constitutes moiré phases in this material. Also, I cannot find the explicit connection between the charge and magnetic orders discussed in this work and the moiré phases. If these points are not discussed well, then in my humble opinion, the "moiré" part should be removed from the title and the main text.

Reviewer #2

(Remarks to the Author)
The authors have addressed my previous comments. I am happy to recommend this manuscript for publication in Nature Communications.

Reviewer #3

(Remarks to the Author)
I co-reviewed this manuscript with one of the reviewers who provided the listed reports. This is part of the Nature Communications initiative to facilitate training in peer review and to provide appropriate recognition for Early Career

Researchers who co-review manuscripts.

Reviewer #4

(Remarks to the Author)
The authors have successfully addressed my previous concerns by providing experimental evidence for the QPI . The inclusion of these results, along with their interpretation, has significantly improved the overall quality of the manuscript. Furthermore, the neutron scattering data has further strengthened the work. I am pleased to recommend the paper for publication in Nature Communications.

Version 3:

Reviewer comments:

Reviewer #1

(Remarks to the Author)
The authors have addressed my previous comments. I am happy to recommend this manuscript for publication in Nature Communications.

Open Access This Peer Review File is licensed under a Creative Commons Attribution 4.0 International License, which permits use, sharing, adaptation, distribution and reproduction in any medium or format, as long as you give appropriate credit to the original author(s) and the source, provide a link to the Creative Commons license, and indicate if changes were made.
In cases where reviewers are anonymous, credit should be given to 'Anonymous Referee' and the source.
The images or other third party material in this Peer Review File are included in the article’s Creative Commons license, unless indicated otherwise in a credit line to the material. If material is not included in the article’s Creative Commons license and your intended use is not permitted by statutory regulation or exceeds the permitted use, you will need to obtain permission directly from the copyright holder.
To view a copy of this license, visit https://creativecommons.org/licenses/by/4.0/

Dear Referees

We thank the referees for their careful evaluation of our manuscript and for their constructive and insightful comments. We apologize for the delay in resubmission, which was due to substantial revisions, including additional data acquisition and relevant analysis/interpretation. In response, we have substantially strengthened the manuscript, both experimentally and conceptually. In light of these advances, we have also adopted a new title—***"Versatile electronic moiré phases enabled by intertwined multiple frustrations in antiferromagnetic two-dimensional semimetals"***—for better capture of the major scope in the revised work. The significant revisions, based on major comments raised by the referees, can be summarized as follows:

1. Additional evidence for frustrating Fermi-surface instabilities by QPI

Most notably, we have introduced comprehensive quasiparticle interference (QPI) measurements, together with detailed analyses, that provide direct evidence for frustrating Fermi-surface instabilities. These results provide a consistent picture across both real and momentum space, significantly reinforcing the experimental foundation of our conclusions. The notable additions are in **Fig. 5**, its related discussion, **supplementary note 6**, and **Fig. S11**. Thanks to the review process, additional/updated detailed temperature (**Fig. 2j**) and B-field dependent investigation (**Fig. 3h**) have uncovered an additional magnetically intertwined charge order, named $CDW3_{mag}$, thereby completing the physical picture presented in the revised manuscript.

2. Deepening discussion at the microscopic level

Based on recent neutron-scattering results at zero magnetic field (summarized at the end of this file, **pages 21~22, and Fig. R2 in this response file**), submitted in a separate study (Ref-50), we have added a detailed discussion clarifying the relationship between the charge and magnetic propagation vectors. Also, a discussion of the mechanism underlying the unusually large energy scale (± 30 meV) observed in the electronic reconstruction is added. We now address this point by discussing how many-body effects—such as inter-pocket Coulomb interactions and momentum-dependent electron–phonon coupling—can naturally give rise to such a large energy scale, going beyond a simple Kondo-driven picture. This discussion will inspire many "future" studies of theoretical considerations.

3. Suitability for Nature Communications

Our results uniquely establish $CeTe_3$ as a versatile platform for realizing multi-q antiferromagnetic charge orders, accompanied by large-energy-scale electronic reconstruction characteristic of strongly correlated systems. This behavior arises from the intertwining of multiple frustration channels—antiferromagnetism, charge ordering, and competing Fermi-surface instabilities—in a semimetallic state. More broadly, our new information supports that antiferromagnetic two-dimensional semimetals can host versatile correlated electronic moiré phases emerging from competing instabilities, going beyond the conventional paradigm of fixed moiré lattices. These findings position van der Waals antiferromagnetic semimetals as a powerful platform for exploring tunable quantum phases in which topology and strong correlations are intrinsically intertwined in two dimensions, while also providing guiding

principles for designing quantum materials with amplified electronic responses to modest external stimuli. We believe that this framework of intertwined frustrations and emergent electronic moiré phases will be of broad and timely interest to the Nature Communications readership.

4. Data reliability and caring setpoint effect

All data presentations have been comprehensively revised throughout the manuscript in response to the referee's comments. These revisions involved substantial and collective improvements in data presentation, while the main scientific conclusions remain unchanged from the initial submission. Concerns regarding experimental procedures—including set-point effects, reproducibility, and the orientation of the applied magnetic field—have been carefully addressed and fully clarified. With respect to set-point effects, although different setpoints were used in some measurements, the key conclusions do not rely on direct comparisons between datasets acquired under different setpoint conditions. Instead, all essential physical trends are extracted from datasets obtained under unified setpoint conditions, ensuring that each dataset provides a self-consistent and reliable basis for the conclusions of this manuscript.

Main manuscript and supplementary

- Major revisions are highlighted in blue.
- Numerous additional minor revisions were made to improve clarity, logical flow, and conciseness; these do not alter the scientific content or conclusions.

Response file

- To minimize the referees' burden, we avoid reproducing large portions of the revised manuscript or figures unless essential, focusing instead on the underlying logic guiding the revisions.
- The relevant revised sections are explicitly indicated.
- Figures referred to as **Fig. 1–5** and **Fig. S1–S11** correspond to those in the revised manuscript and revised **Supplementary Information**. The figure shown in this response is only **Fig. R1**.
- We submit neutron data as a separate file named "*position of neutron data*".

We apologize for any deviation from a conventional response format and hope this structure facilitates a clear, efficient review of the revisions without placing an undue burden on the referee. Below, we address each point in the comments point by point.

Sincerely,

Yoshinori Okada (on behalf of all authors)

Associate Professor, Okinawa Institute of Science and Technology (OIST), Japan

Reviewer #1 (Remarks to the Author):

The manuscript Versatile multi-q antiferromagnetic charge order in correlated vdW metals by Fujisawa, et al. presents STM data on the layered antiferromagnet CeTe3. The main findings of this work are

1) Appearance of the stripe charge order across magnetic ordering temperature (Neel temperature)

2) Appearance of the checkboard charge order and suppression of the stripe charge order across spin flop transition.

Discovery of charge orders intertwined with magnetic order is one of the exotic phenomena in condensed matter physics. Also, the manuscript is written clearly and is well-organized. In principle I am in favor of recommending this paper for publication if authors successfully address my concerns and comments below.

We appreciate the referee for his/her time for reviewing and providing positive and constructive comments.

Major comments

1) Further insights on underlying mechanism of intertwining: It would be much more insightful and impactful if the authors characterize microscopic structure of the underlying magnetic ordering below Neel temperature and above spin-flop magnetic field (and propose underlying mechanism of intertwined charge and magnetic order + competition between CDW2_mag and CBC_1,2). In this regard, would it be possible for authors to conduct spin-polarized STM as in study of other RTe3 system [npj Quantum Mater. 9, 47 (2024)]? I understand it could be a long journey so If the authors have no previous experience of spin-polarized STM, this comment can be skipped over.

Following the referee's comment, we have incorporated a discussion based on available neutron-diffraction results. As these measures are still ongoing, we restrict our discussion to conclusions that are firmly established at this stage. While we appreciate the referee's suggestion, we respectfully disagree that SP-STM/STS is necessary at the present stage. More details are summarized below.

<u>The relation between magnetic q_m and charge q_c, and the microscopic mechanism</u>

Based on the magnetic reflections described above, we identify a possible physical mechanism connecting the magnetic propagation vector q_m to the charge-ordering vector q_c. This relation is consistent with recent theoretical models incorporating Kondo exchange coupling. Confirmation of this relation at zero field further supports the emergence of a distinct magnetic order above B_{flop}, assuming a comparable exchange coupling strength. Thus, although detailed magnetic-structure information in the spin-flop phase is not yet available, our results suggest the presence of a distinct magnetic structure in this field regime.

<u>Spin-polarized STM</u>

Spin-polarized STM under a varying magnetic field inevitably convolutes intrinsic changes in the sample density of states with field-dependent spin-dependent tunneling matrix elements, even in the absence of a genuine DOS modification. For this reason, we deliberately relied on systematic STM/STS measurements using a non-magnetic tip. This choice is particularly important for $CeTe_3$, where the band structure itself evolves strongly with magnetic order and applied field. In contrast to other RTe_3 compounds, where magnetism-driven band deformation is relatively

weak, STM/STS with a non-magnetic tip is essential to disentangle intrinsic electronic reconstruction from tunneling-related effects and to ensure an unambiguous interpretation of the field dependence. While SP-STM/STS is an important direction for future work, we consider it more appropriate as an independent follow-up study rather than part of the already massive present manuscript.

Related revisions:

- In the *introduction* part, the rationale for not using SP-STM is clarified.
  Corresponding sentences are *"All measurements employed a non-magnetic STM tip, enabling direct access to intrinsic DOS reconstructions across T_N and B_{flop} without convolution from spin-dependent tunneling matrix elements inherent to spin-polarized STM."*
- In the *results and discussion* part, to deepen the discussion on the mechanism of the microscopic picture, the following sections are added or significantly revised
  - ✓ ***Quasiparticle interference pattern***
  - ✓ ***Magnetic propagation vectors and spin–charge intertwining***
  - ✓ ***Underlying microscopic interactions***

2) Novelty: Several recent publications showcase the intertwining of magnetic and electronic orders in RTe3 materials. One example is from X-ray and neutron studies of TbTe3, published in Chillal, et al., Phys. Rev. B 102, 241110(R) (2020). Another example includes CeTe3 itself, as seen in a magnetotransport study published in Higashihara, et al., Phys. Rev. B 109, 134404 (2024). In the paper by Higashihara, et al., the resistivity and carrier concentration in CeTe3 are shown to change abruptly below the magnetic transition, while remaining relatively steady in TbTe3. Although STM visualization of changes in density-of-states maps demonstrated in the present work does provide further information, could the authors show more directly how the present work yields novel insights justifying publication in Nat. Commun.?

We thank the referee for pointing out these relevant previous studies, cited as ref. 29 and 43 in the revised manuscript. While these studies have suggested underlying spin-charge interplay, they cannot resolve how competing electronic instabilities reorganize the low-energy electronic structure in real or momentum space.

Notably, The decisive advance of the present work is the introduction of comprehensive quasiparticle interference (QPI) measurements together with detailed analyses, which provide direct and unambiguous evidence for Fermi-surface reconstruction, consistently viewed from both real-space and momentum-space perspectives. These additions significantly strengthen the experimental foundation of our conclusions. Moreover, prompted by the review process, we have identified an additional magnetically intertwined charge order, $CDW3_{mag}$, thereby completing a coherent and self-consistent physical picture in the revised manuscript. Beyond the specific case of $CeTe_3$, our spectroscopy-based results establish a broadly applicable and transferable framework for identifying multi-q intertwined electronic states rooted in Fermiology. This framework is readily extendable to a wide class of antiferromagnetic van der Waals metals and semimetals, positioning the present study as a general paradigm for exploring multi-q correlated states

and emergent phases in two-dimensional quantum materials—an area of timely and central interest in current quantum materials research. Overall, with the inclusion of momentum-resolved QPI measurements and a clarified discussion of the underlying microscopic interactions, we believe that the revised manuscript represents a substantial and timely advance and is well-suited for publication in *Nature Communications*.

Related revisions:

- In the *introduction* part, to highlight how our manuscript goes beyond existing literature, the following sections have been added or significantly revised
  - ✓ ***Significant position of CeTe3***
  - ✓ ***Position of this study***
- In the *results and discussion* part, to deepen the discussion on the mechanism of the microscopic picture, the following sections are added or significantly revised
  - ✓ ***Quasiparticle interference pattern***
  - ✓ ***Magnetic propagation vectors and spin–charge intertwining***
  - ✓ ***Underlying microscopic interactions***
  - ✓ ***Outlook and future perspective***

3) Data repeatability: Having looked at both the main text and the supplementary material, the presented data appears to have come from a single set of measurements. The sample was measured at 4 K, it was then cooled down to 300 mK, and then an in-plane magnetic field up to 2.5 T was ramped up in increments while the sample was held at 300 mK. For a journal of Nat. Commun.'s caliber showing repeatability would make the arguments a lot more convincing. For example, did the Fourier intensities match when the field was ramped back down to 0 T while at 300 mK? It has been shown, for example, in Fu, Kraft, et al., Phys. Rev. B 94, 205101 (2016), that STM measurements of Fourier intensities in RTe3 materials can be highly sensitive to tip conditions. Given this, data addressing repeatability would make the arguments much more watertight.

While STM/STS provides a powerful probe of low-energy electronic structure, we fully agree that this technique requires particular attention to ensure data reliability. In this study, the reproducibility of all STM results underlying our main conclusions was carefully verified using multiple tips and more than ten independently cleaved sample surfaces. Any detectable change in tip condition or surface quality was treated as grounds to discard the affected data and restart the measurement procedure. In addition, special attention was paid to possible drift induced by changes in the magnetic field and temperature. All interpretations presented in this study are based exclusively on such internally consistent/reproducible datasets.

Drift and tip/surface conditions

To ensure the data reliability of **Fig. 3h**, we include raw STM topographs acquired immediately before and after the

magnetic-field-dependent measurements, together with their corresponding Fourier-transformed images (**Supplementary Note 4**). These data demonstrate that both the tip condition and the surface morphology remain essentially unchanged throughout the experiment. Importantly, these confirm that the measurements were performed on the same spatial area before and after the magnetic-field sweep. We further confirm that the Fourier intensities measured at B = 0 T after ramping the magnetic field up to 2.5 T and back down at 300 mK are consistent with those obtained before the field-dependent measurements. Although a small variation in the FFT intensity at B = 0 T is observed before and after the field cycle, its magnitude is minor and does not affect any of the conclusions drawn in this work. Investigation of possible intrinsic hysteresis effects is an interesting direction for future study; however, we consider this beyond the scope of the present manuscript and more appropriately an independent follow-up investigation.

Related revisions:

- About data reliability, the corresponding revised part is in **Supplementary Note 4.**

Minor comments

1) All the dI/dV spectra shown are between -30 mV and +30 mV or so. Can the authors show 1-2 larger energy range spectra as well? It would be great to include dI/dV comparison for a wider range of energy to show from which energy the DOS across T_N starts to converge.

We appreciate the referee's insightful comment regarding the energy window used in the main text. In response, we performed additional STM/STS measurements over an extended energy range up to ±90 meV (**Fig. 1g**). The resulting spectra show that the density of states outside the ±30 meV window exhibits only minimal variation across T_N and under applied magnetic field, whereas the pronounced spectral reconstruction is confined to the energy range highlighted in the main manuscript.

2) Missing setpoint information in Fig.3e

We apologize for the incomplete information regarding the experimental condition. We have included the set bias and set current. It is +50 meV and 2.5 nA, respectively. We have added the information in the figure caption. We believe that this comment should be considered throughout the manuscript. Accordingly, we have applied the same level of care across the entire paper and thoroughly revised all relevant sections.

3) Regarding spin-flop phase, can author present multiple energy slices of the dI/dV map so that one can check at which energy the checkerboard pattern of CBC1,2 is most prominent? Also, can the author shows dI/dV map at a fixed energy (maybe at -6 meV as the CDW2_mag was most clearly visualized?) with the magnetic field below and above spin-flop transition, so that one can visually see the competition of CDW2_mag and CBC in real space.

We appreciate the referee's comments regarding the presentation of dI/dV maps at different energies above B_{flop}. Regarding the energy dependence, we now present the relevant data in the revised **Fig. 3c**. In the revised manuscript, all relevant information has been provided to make it as transparent as possible. Please note that, thanks to the reviewing process, we have a $CDW3_{mag}$ to complete a full story.

4) It is stated in the manuscript that all dI/dV spectra are normalized by the area between 0 mV and -30 mV. It would be useful to also see a comparison with spectra which are not normalized using this procedure.

Based on the referee's comments, we have shown the raw dI/dV data in **Fig. S7b**, demonstrating that the normalization procedure doesn't change the main conclusion of this study.

In summary (for the editor), I recommend publishing this work if the authors address my comments above.

We believe that we have carefully and comprehensively addressed all the referee's concerns, and thus the manuscript merits publication in *Nature Communications*.

Reviewer #2 (Remarks to the Author):

By using scanning tunneling microscopy and spectroscopy at 300 mK, the authors uncover two competing states in vdW metal $CeTe_3$: charge-ordered in-plane antiferromagnetic phases forming stripe and checkerboard patterns. Remarkably, the competition between them is tuned through a modest in-plane magnetic field (~1.5 T), revealing significant cooperative phenomena between frustrated antiferromagnetism, charge order, and competing Fermi surface nesting. While the results are interesting, however, some important issues need to be addressed before considering the publication in nature communications.

We appreciate the referee for his/her time for reviewing and providing constructive comments.

1. Figure 1b shows for CeTe3, no CDW2 phase exists; and TN is about 1 K, which is consistence with Fig.1h. However, in Fig.2, the author observed CDW2mag at 300 mK. Does it mean the refence 26 is wrong, or just because not going lower temperature? Is CDW2mag is same as CDW2?

We greatly appreciate this comment. We fully admit our presentation needs to be properly revised. Following the referee's comment, we have revised the introduction extensively to avoid any misleading/incorrect statements. To be short, two points from the literature are clearly written.

1. Literature 26 (currently 23) does not simply recognize $CDW2_{mag}$, presumably, not accessible to the corresponding low temperature. So, the literature is not necessarily wrong.
2. While the other previous literature indeed pointed out the existence of a distinct density wave order from specific heat and transport (ref. 17 and 48), our current study has uniquely uncovered a microscopic picture using STM/STS.

Related revisions:

- In the introduction, the corresponding revised part is the sections
  - ✓ ***Significant position of $CeTe_3$***
  - ✓ ***Position of this study***

The authors state "Although its nondispersive wavevector (0.33, 0) resembles CDW2 found in the other RTe_3 compounds, its exclusive onset below TN points to a different mechanism, justifying the term CDW2mag due to its connection to magnetism." The onset below TN cannot justify the CDW2mag due to magnetism, it can be other mechanism, for example the evolution of phonon spectrum. The author needs more evidence to clarify the magnetic origination of CDW2.

We thank the referee for this comment. To be short, by adding a temperature-dependent study (**Fig. 2j**) and deepening the discussion relying on neutron data, we have further convinced that $CDW2_{mag}$ (and CBC_{mag}) is magnetically

intertwined. Further details are as follows.

Magnetic intertwined charge order $CDW2_{mag}$ and CBC_{mag}

Based on the following, we believe our argument on intertwined antiferromagnetism on the formation of $CDW2_{mag}$ and CBC_{mag} is much more strongly supported.

1. First, the onset of $CDW2_{mag}$ occurs only below the T_N (**Fig. 2j**).
2. We don't see analogous charge order down to 300mK (minimum temperature we can access) in $LaTe_3$ (our previous paper PRB 110, 235415 (2024))
3. Based on Kondo coupling, the neutron data points $CDW2_{mag}$ are indeed magnetically coupled.
4. The wavevector associated with $CDW2_{mag}$ exhibits a clear evolution across the spin-flop transition, directly indicating coupling to the magnetic degrees of freedom.

Therefore, while we sincerely consider the referee's comment, we still believe $CDW2_{mag}$ (and CBC_{mag}) is magnetically intertwined. On the other hand, for the newly uncovered $CDW3_{mag}$, the connection to neutron data is unclear at this time. In this regard, we have widely discussed the origin of $CDW3_{mag}$.

Nevertheless, this does not necessarily exclude consideration of additional factors. From a broad perspective, we have discussed various possibilities, including electron-phonon and electron-hole coupling, to realize a versatile antiferromagnetic charge order accompanied by a wide-range energy-scale renormalization (±30 meV). This richly intertwined phenomenology is exactly what we would like to convey as the main message of the manuscript.

Related revisions:

- ➢ We have clarified the terminology for how we use the subscript "mag".
  Corresponding sentence is "*Throughout this work, the subscript "mag" is used to indicate charge orders that appear exclusively within magnetically ordered states.*"
- ➢ In the Results and Discussion part, to highlight how our manuscript goes beyond existing literature, the following sections are revised or added.
  - ✓ ***Emergence of magnetically driven charge order across T_N***
  - ✓ ***Field-induced emergence of CBC_{mag} above F_{lop}***
  - ✓ ***Field-tuned competition of antiferromagnetic charge orders***
  - ✓ ***Magnetic propagation vectors and spin–charge intertwining***
  - ✓ ***Underlying microscopic interactions***

2. Please note what the blue arrows in Fig. 2e stand for. And what is the abbreviation of "r.l.u."? and also please mark the pathway of line-cut profile of the measurement of Fig. 2f in Fig. 2d. If assuming the (0.0) position is for the

central spot, what are the rest of three spots in panel d? And the label of horizontal axis in 2f is missing.

We thank the referee for pointing out these incomplete descriptions. Since the figures have been extensively revised, the revision linked to the comment is no longer relevant. However, throughout the overall manuscript, we have carefully and sincerely considered these comments and have revised the text to minimize any similar improper or insufficient descriptions.

Newly uncovered $CDW3_{mag}$

In particular, let us address our response to the question on peaks around (0,0) in *old* **Fig. 2d** and *old* **Fig. 3b** since this needs relatively careful explanation. While we noticed these in the previous version (assigned as q* in the *old* **supplementary note 1**), we have positioned q* as the minor role in drawing the main conclusion of the manuscript. However, thanks to the referee's comment, we were able to assign q* as $CDW3_{mag}$. Most notably, $CDW3_{mag}$ is now collectively interpreted with $CDW2_{mag}$ and CBC_{mag} in the revised manuscript (e.g., in **Fig. 1j**, **Fig. 2j**, **Fig. 3h**, and **Fig. 5**).

Related revisions:

- With care, we have prepared all of the revised Figures.
- In the Results and Discussion part, to highlight how our manuscript goes beyond existing literature, the following sections are revised or added.
  - ✓ ***Emergence of magnetically driven charge order across T_N***
  - ✓ ***Field-induced emergence of CBC_{mag} above B_{flop}***
  - ✓ ***Field-tuned competition of antiferromagnetic charge orders***
  - ✓ ***Magnetic propagation vectors and spin–charge intertwining***

3. Fig. ~~3~~2g shows the energy dependence of the peak intensities associated with CDW2mag relative to the Bragg peak. However, it doesn't consistence with Fig. 2f, I don't see the intensity peak at +15 meV in f panel. please explain.

We thank the referee for pointing out this. We clarify that the previous **Fig. 2g** was obtained under a positive set-point condition (+50 mV / 2.5 nA). Under this condition, the dI/dV signal at positive bias is intrinsically weaker than that at negative bias, as seen in **Fig. 2f.** This asymmetry primarily originates from the well-known set-point effect, whereby the dI/dV contrast at energies with the same sign as the set bias tends to be suppressed. These potential confusions stem from the setpoint effect, and we revised the presentation to avoid confusion or misrepresentation in the revised manuscript.

Related revisions:

- In the Results and Discussion part, to give transparent logic to select particular bias voltages in taking data set

in **Fig. 2, 3, and 4**,

4. Is there any particular reason to compare the line-cut profile of the Fourier signal at ±22 meV in Fig. 2h?

We agree that the rationale for this data presentation was not sufficiently explained and may have caused confusion. Previously, the original choice of ±22 meV was motivated by the fact that the $CDW2_{mag}$ signal is most clearly resolved around this energy. Following this comment, we have removed these plots from the revised manuscript to avoid confusing readers. We believe that the main logic is clearer.

5. Currently, the FT image in Fig. 3b is taken from the topographic image of a panel. Why not take from the dI/dV mapping of the d panel, which at least could remove the topographic contribution. Actually, all topographic images are look very similar. The lattice of surface Te contributes to the image mostly, while the electronic DOS contributes a little.

As the referee correctly points out, dI/dV maps generally suppress contrast originating from surface topography more effectively than STM topographs. Performing field-dependent measurements using complete dI/dV mappings is substantially more time-consuming than acquiring topographs, making systematic measurements across many magnetic fields impractical. It is also important to emphasize that such a dataset should be obtained without any change in the tip-sample tunneling condition. We therefore used FFTs of topographic images for the field-dependent analysis. For this reason, **Fig. 3b** presents the FFT of a topograph, ensuring consistency with the dataset used to construct **Fig. 3e**. We believe this approach is both appropriate and reasonable for capturing the magnetic-field evolution of the Fourier intensity, and we have added discussion in the manuscript to clarify and justify this choice.

Related revisions:

- The rationale for using the topographic image is provided in the **Method** section and **Supplementary note 2**.

6. A critical concern is, all topographic images of Fig. 1g, Fig. 3a, the Fig. 2a and 2c, the measuring setpoints are different. Thus the distance of tip/sample are different, may cause some tip effect on the imaging. The authors need exclude such effect.

We appreciate the referee's careful attention to the measurement conditions. Although the setpoints used in some data differ, we emphasize that the main text's key message does not rely on direct comparisons between datasets acquired under different setpoint conditions. As long as the datasets used to extract essential physical information are obtained under unified setpoint conditions, reliable trends in the stabilization and destabilization of the antiferromagnetic charge orders can be established. For example, each dataset shown in **Fig. 2j**, **Fig. 3h**, and **Fig. 4a–d** was acquired under identical internal measurement conditions, although the absolute setpoint differs between datasets depending on the specific experimental scope. Consequently, each dataset provides a self-consistent and reliable basis for constructing the main conclusions of this manuscript.

Related revisions:

➢ The relevant details are provided in the **Methods** section and **Supplementary Note 2**.

7. How is the spatial evolution of dI/dV spectra? When the author states the Fig. 4a shows evolution of the spatially averaged dI/dV spectra under in-plane magnetic fields, please clarify how the measurement are performed and how spatially average being done.

We thank the referee for raising this point and acknowledge that this necessary clarification was not sufficiently described in the previous version. While spatial variations in dI/dV intrinsically arise from defects, impurities, and QPI, we have carefully accounted for these effects by performing proper spatial averaging over sufficiently large areas. This procedure ensures that the reported spectral features reflect intrinsic electronic reconstruction rather than local inhomogeneities.

Related revisions:

➢ In **Method**, we have included a detailed process for spatially averaging the dI/dV spectra.
➢ In **Supplementary Note 3**, we have included the spatial variation of the dI/dV spectra.

8. Most importantly, the author claims the observed CDW2mag and CBCmag are due to magnetic orders, however, there is no direct evidence to support it. What the author have is the B dependence of ICDW2/IBragg and ICBC1,2/IBragg. It is better to use spin-polarized STM technique to resolve the related spin texture.

While we appreciate the referee's suggestion, we respectfully disagree that SP-STM/STS is necessary at the present stage. More details are summarized below.

Spin-polarized STM

Spin-polarized STM under a varying magnetic field inevitably convolutes intrinsic changes in the sample density of states with field-dependent spin-dependent tunneling matrix elements, even in the absence of a genuine DOS modification. For this reason, we deliberately relied on systematic STM/STS measurements using a non-magnetic tip. This choice is particularly important for $CeTe_3$, where the band structure itself evolves strongly with magnetic order and applied field. In contrast to other RTe_3 compounds, where magnetism-driven band deformation is relatively weak, conventional STM/STS is essential to disentangle intrinsic electronic reconstruction from tunneling-related effects and to ensure an unambiguous interpretation of the field dependence. While SP-STM/STS is an important direction for future work, we consider it more appropriate as an independent follow-up study rather than part of the present manuscript.

The relation between magnetic q_m and charge q_c, and the microscopic mechanism

Based on the magnetic reflections described above, we identify a physical mechanism connecting the magnetic propagation vector q_m to the charge-ordering vector q_c. This relation is consistent with recent theoretical models

incorporating Kondo exchange coupling. Confirmation of this relation at zero field further supports the emergence of a distinct magnetic order above B_{flop}, assuming a comparable exchange coupling strength. Thus, although detailed magnetic-structure information in the spin-flop phase is not yet available, our results suggest the presence of a distinct magnetic structure in this field regime.

Magnetic intertwined charge order $CDW2_{mag}$ and CBC_{mag}

Based on the following, we believe our argument on intertwined antiferromagnetism on the formation of $CDW2_{mag}$ and CBC_{mag} is much more strongly supported.

- First, the onset of $CDW2_{mag}$ occurs only below the T_N (**Fig. 2j**).
- We don't see analogous charge order down to 300mK (minimum temperature we can access) in $LaTe_3$ (our previous paper PRB 110, 235415 (2024))
- Based on Kondo coupling, the neutron data points $CDW2_{mag}$ are indeed magnetically coupled.
- The wavevector associated with $CDW2_{mag}$ exhibits a clear evolution across the spin-flop transition, directly indicating coupling to the magnetic degrees of freedom.

Related revisions:

- Mechanism of intertwining is deeply discussed with our best at the current stage in the sections
  - ✓ ***Quasiparticle interference pattern***
  - ✓ ***Magnetic propagation vectors and spin–charge intertwining***
  - ✓ ***Underlying microscopic interactions***
- In the introduction, the rationale for not using SP-STM is clarified as a significant first step.
  Corresponding sentences are *"All measurements employed a non-magnetic STM tip, enabling direct access to intrinsic DOS reconstructions across T_N and B_{flop} without convolution from spin-dependent tunneling matrix elements inherent to spin-polarized STM."*

9. Magnetic field of 1.5 T induce a spin-flop, the author assume that a net ferromagnetic component emerges from the AF background at magnetic fields above Bflop. While the author attributes the competing nature of CDW2mag and CBCmag states to small-perturbation driven wide energy range deformations of DOS around EF. As we know, Bflop (~1.5 T) and TN (~1.5 K) is a relatively small energy, I doubt it can drive a FS deformation. The author gives many possible mechanisms, such as via Zeeman-induced band splitting, a spin-orbital effect or electron-phonon coupling. The author need provide a confirmative speculation with solid evidence.

With respect to this concern, we emphasize that the use of a non-magnetic STM tip is essential to the validity of the present conclusions and, in this context, provides a decisive advantage over spin-polarized STM. The STM tip condition was rigorously monitored and verified to remain unchanged throughout the entire data acquisition, fully consistent with established best practices in the STM/STS community. Accordingly, the observed spectral

reconstruction extending to ±30 meV cannot be attributed to any tip-related artifacts. Moreover, because the tip is non-magnetic, spin-dependent tunneling matrix-element effects are categorically excluded, leaving intrinsic changes in the sample's electronic structure as the only possible origin of the observed dI/dV variations. Furthermore, the Fermi surface deformation is experimentally visualized using the QPI technique in the revised manuscript. We therefore conclude unambiguously that the ±30 meV reconstruction represents an intrinsic electronic response of $CeTe_3$.

Mechanism: why small energy scales induce competing charge orders in $CeTe_3$

We agree that the bare energy scales associated with the spin-flop field B_{flop} (~1.5 T) and the Néel temperature T_N (~1.5 K) are small compared to the ±30 meV energy window over which pronounced DOS reconstruction is observed; however, this apparent disparity does not represent an inconsistency. Rather, it directly indicates the involvement of additional interactions beyond simple magnetic energy scales. In the revised manuscript, we explicitly discuss how Coulomb interactions between small electron and hole pockets and momentum-dependent electron–phonon coupling can naturally give rise to large electronic energy scales, even when the magnetic ordering energies are modest. At the same time, we have deliberately removed speculative elements present in the earlier version to ensure that the discussion remains physically grounded and tightly constrained by the experimental observations.

Related revisions:

- In the Results and Discussion part, the following section is added/revised
  - ✓ ***Underlying microscopic interactions***

Overall, I think the experimental results is not enough to support their claim. Therefore, I suggest the author need provide more solid experimental evidence to claim the observation of multi-q antiferromagnetic charge order in CeTe3.

We believe that the addition of detailed temperature-dependent measurements, comprehensive QPI analysis, and an expanded discussion of the microscopic mechanism, together with neutron data, collectively strengthens the manuscript and enhances its merit for publication in *Nature Communications*.

Reviewer #3 (Remarks to the Author):

I co-reviewed this manuscript with one of the reviewers who provided the listed reports. This is part of the Nature Communications initiative to facilitate training in peer review and to provide appropriate recognition for Early Career Researchers who co-review manuscripts.

Reviewer #4 (Remarks to the Author):

The authors present STM/STS measurements on the cleaved surface of the van der Waals compound CeTe3, conducted over a temperature range from 4 K to 300 mK and under in-plane magnetic fields up to 2 T. Fourier transforms of both the topography and the dI/dV maps reveal a CDW with wave vector q = (0, 0.28) in the normal state at 4 K. At lower temperatures, below the antiferromagnetic transition, additional periodicities appear: q = (0.33, 0) at zero field and q = (0.19, +-0.19) under in-plane fields exceeding the spin-flop transition. These additional charge orders are discussed in relation to nesting vectors on a proposed phenomenological Fermi surface. The manuscript also includes STS data across the AF phase, both above and below the spin-flop transition. The experimental results and the interpretation are generally reasonable. However, the absence of quasiparticle interference measurements or electronic band calculations is a significant omission. Without these, it is difficult to convincingly disentangle the interplay between charge and magnetic orders in CeTe3. Additionally, the manuscript lacks a strong connection to the more exotic or topological multi-q phases referenced in the abstract and introduction. For these reasons, I believe the work may be more appropriate for a specialized journal rather than Nature Communications.

We thank the referee for the time and care devoted to reviewing our manuscript and for the constructive comments. In direct response to this first point, we have performed comprehensive quasiparticle interference (QPI) measurements that directly visualize the Fermi-surface reconstruction associated with the $CDW2_{mag}$ and CBC_{mag} phases. Motivated by the referees' suggestions, we have also identified an additional magnetically intertwined charge order, denoted $CDW3_{mag}$. Importantly, the coexistence and competition among $CDW2_{mag}$, $CDW3_{mag}$, and CBC_{mag} are now consistently and quantitatively interpreted using the newly acquired QPI data. By further linking these observations to the underlying magnetic structure through Kondo exchange coupling, we demonstrate how magnetic reconstruction selectively stabilizes competing Fermi-surface instabilities.

The notable data additions are in **Fig. 5**, its related discussion, **supplementary note 6**, and **Fig. S11**. Thanks to the review process, additional/updated detailed temperature (**Fig. 2j**) and B-field dependent investigation (**Fig. 3h**) have uncovered an additional magnetically intertwined charge order, named $CDW3_{mag}$, thereby completing the physical picture presented in the revised manuscript.

In addition, we have significantly deepened the discussion of both the fundamental and practical implications of our findings, clearly positioning these aspects as a forward-looking perspective of the work. While the emergence of a topological magnetic phase is not directly demonstrated in the present study, we believe that the combination of detailed temperature-dependent measurements, comprehensive QPI analysis, and an expanded discussion of the microscopic mechanism, together with available neutron-scattering information, substantially strengthens the manuscript. Collectively, these additions enhance the conceptual clarity, experimental rigor, and overall impact of the work, making it well-suited for publication in *Nature Communications*.

Related revisions:

- The mechanism of the microscopic picture is deeply discussed with our best at the current stage in the sections
  - ✓ ***Quasiparticle interference pattern***
  - ✓ ***Magnetic propagation vectors and spin–charge intertwining***
  - ✓ ***Underlying microscopic interactions***
  - ✓ ***Outlook and future perspective***
  - ✓ ***Summary***

Comments:

1. Prior studies by the authors (e.g., Fig. 4 in Ref. 11) report two AF transitions at approximately 3 K and 1 K. Does the sample in this study not exhibit a transition at 3 K? The lack of experimental data near 3 K and the absence of discussion regarding this known feature raise concerns about completeness and scientific transparency.

We appreciate the referee's insightful comment regarding the temperature range between T_{N1} and T_{N2}. To avoid confusion, let us first clarify that T_{N1} ~ 3K and T_{N2} ~ 1K were used in the previous study. In this study, we mainly focus on the intertwined state in the long-range antiferromagnetic ordered state below T_{N2}. To clarify this point, we use T_N in the revised main body to denote the onset temperature of long-range antiferromagnetic order (T_{N2}).

We think that the intermediate regime $T_{N2} < T < T_{N1}$ is likely characterized by magnetic fluctuations without the establishment of long-range magnetic order, and based on our very preliminary observations (not shown here), this regime presumably hosts interesting physics. At the same time, the primary objective of the present study is to establish the nature of the *long-range*, versatile antiferromagnetic charge-ordered states stabilized below T_{N2}, which is the essential first step in positioning antiferromagnetic van der Waals semimetals as a robust and broadly relevant quantum-materials platform. Clarifying this ground-state phenomenology—its real-space structure, momentum-space origin, and field-tuned competition—is both conceptually and experimentally the most critical foundation for the community. In this context, we do not consider the current manuscript to be incomplete. Moreover, the volume and depth of the experimental results already presented in the revised manuscript are substantial, and incorporating a detailed investigation of the fluctuation-dominated intermediate regime would significantly broaden the scope beyond a coherent, focused narrative. We therefore believe that a systematic study of the $T_{N2} < T < T_{N1}$ regime is best positioned as separate future work, naturally building on the framework established here. We hope the referee agrees that this delineation strengthens the clarity of the present manuscript.

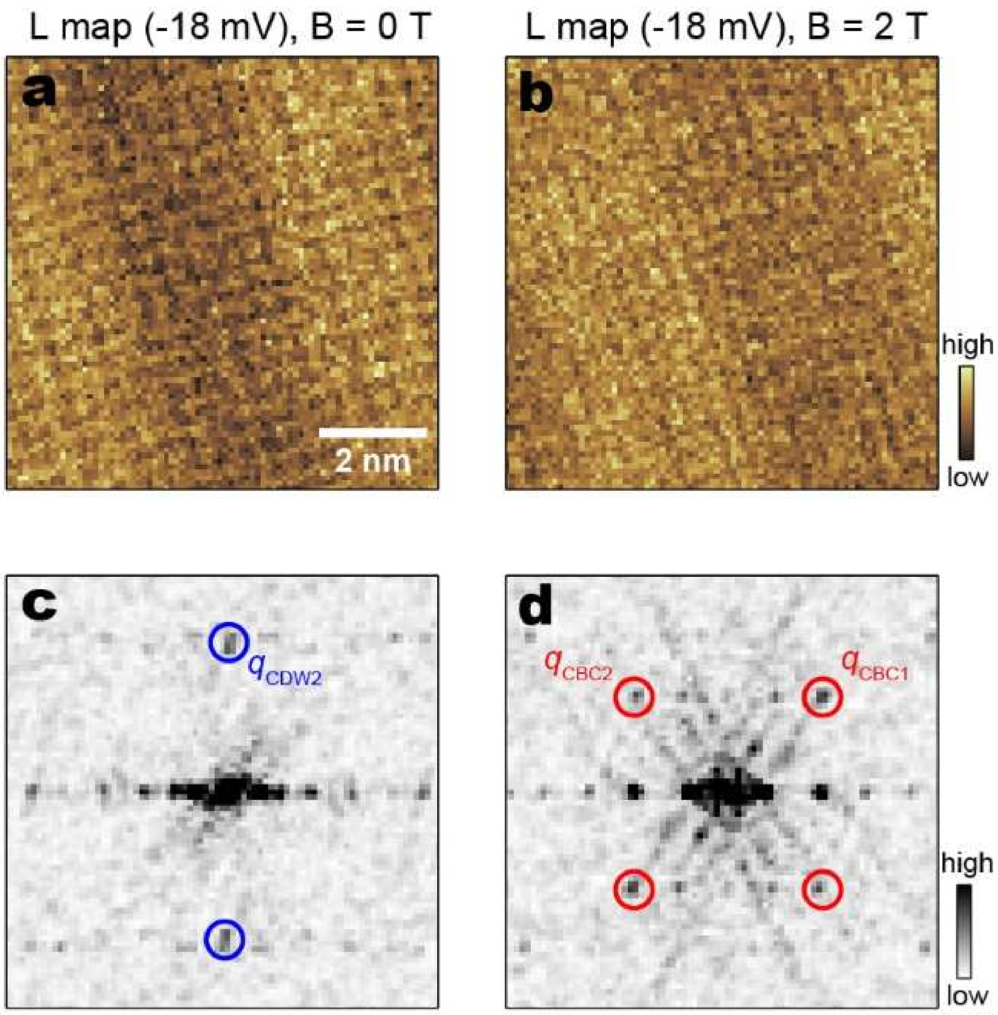

Fig. R1

(a,b) L-maps obtained from the same dataset as Fig. 2e and Fig. 3c, respectively.

(c,d) Their Fourier-transformed images.

Related revisions:

- In the introduction, the main focus on the long-range magnetic ordered state below T_N is clearly stated.
- In the "**Sample growth and characterization**" section of the Methods, the focus of this study on the long-range magnetically ordered state is clearly stated, while studying above T_N is noted as interesting for future study. The major relevant sentences are

  "In the previous report, two characteristic temperatures (T_{N1} and T_{N2}) were pointed out [ref]. T_{N1} corresponds to short-range magnetic fluctuation, and T_{N2} corresponds to long-range antiferromagnetic order. Note that the T_N in this report corresponds to T_{N2} in the literature. "

2. The authors state that they normalize I_CDW by I_Bragg, but the rationale for this is unclear. Wouldn't it be more appropriate to analyze the normalized conductance L=(dI/dV)/(I/V), in order to mitigate the set-point effect? Clarification is needed.

Attempt to the analysis of L-map

We agree with the referee that the normalized conductance L = (dI/dV)/(I/V) is a useful quantity to reduce the set-point effect. In response to this comment, we have calculated the corresponding L maps and their Fourier-transformed images, which are now presented in **Fig. R1**. We find that, while the characteristic wave vectors associated with $CDW2_{mag}$ and CBC_{mag} remain identifiable in Fourier space, their real-space contrast is significantly reduced in the L maps compared to the *dI/dV* maps. We think this is because the energy-independent LDOS modulations at a momentum q tend to be suppressed, because both the denominator (I) and the numerator (dI/dV) contain a sharp peak at q.

Reasoning for using I_{CDW}/I_{Brag}

We have revised **Fig. 3h**, which presents the magnetic-field dependence of the FFT intensities of the charge orders normalized by the corresponding Bragg peaks. We now explicitly clarify that this normalization is not intended to compare absolute FFT intensities between different charge orders, but rather to suppress small anisotropic contrast arising from the STM tip. For transparency, the raw, unnormalized data are provided in **Fig. S5**. Importantly, both normalized and raw datasets consistently demonstrate the central conclusion of this study: the pronounced field-tuned competition between $CDW2_{mag}$ and CBC_{mag}.

3. What is the orientation of the applied in-plane magnetic field with respect to the crystal axes? The discrepancy in the spin-flop field between magnetization and STM measurements suggests that the field may not lie strictly along the crystallographic axes (e.g., a deviation from the c-axis in the ac plane). This ambiguity makes it difficult to interpret the STM results in the context of the underlying magnetism. It is important to experimentally rule out the possibility that the observed features arise from the misalignment of the magnetic field.

We thank the referee for raising this important point regarding the orientation of the applied in-plane magnetic field. To eliminate the possibility that our observations arise from magnetic-field misalignment, we performed a calibration measurement using $NbSe_2$ prior to the experiments on $CeTe_3$. In $NbSe_2$, it is well established that a tilted magnetic field generates superconducting vortices with elongated tails whose orientation directly tracks the direction of the in-plane field component. As shown in the newly added Supplementary **Fig. S11**, the orientation of these vortex tails is fully consistent with the nominal field direction in our setup. This confirms that the in-plane magnetic field was applied accurately and that any misalignment, if present, is negligible. We therefore believe that the features reported in the manuscript do not originate from field-alignment issues, but from the small difference in the measurement temperature.

Related revisions:

- We have added a **Supplementary Note 5** to establish that our magnetic field direction is well-calibrated.

4. The set-point parameters for the 4 K and 0.3 K data in Fig. 1f appear inconsistent. These should be aligned ideally.

We thank the referee for pointing out the difference in the setpoint for old-**Fig. 1f** (revised-**Fig. 1g**), representing the variation in the spectra across T_N. We performed an additional experiment to demonstrate that the dataset was collected under identical conditions.

Related revisions:

- We have revised **Fig. 1g** to show the temperature dependence of the dI/dV spectra at an identical setpoint.

5. It is unclear which "l" the line cut in Fig.2f is at.

We now draw a red arrow in **Fig. 2d** to show which momentum direction is shown in **Fig. 2f**.

Position of Neutron Experiments

A manuscript reporting the neutron diffraction study is currently under review at Physical Review B (cited here as Ref. [50]), entitled "Quantum fluctuation–driven double-q magnetic order in the van der Waals metal $CeTe_3$" by R. Okuma, Y. Fujisawa, N. Maekawa, A. Nakao, Y. Ishikawa, R. Kobayashi, Y. Okada, and D. Ueta.

Here, we summarize only the aspects of the neutron results that are directly relevant to the present study. The neutron diffraction study constitutes an independent and complementary investigation that determines the zero-field magnetic structure of $CeTe_3$, whose propagation vectors provide the necessary context for interpreting the STM observations.

In the present manuscript, the primary focus is the magnetic-field-induced switching of intertwined charge-ordered states revealed by STM/STS measurements, together with the identification of its Fermi-surface origin through quasiparticle interference (QPI) analysis. These results demonstrate that the field-induced electronic reconstruction and its Fermi-surface origin are fully established by the STM and QPI measurements presented in this work. The distinct scientific focus of the two studies naturally leads us to present them as separate manuscripts.

Neutron-scattering results at zero magnetic field

Figure R2(a) shows the scattering intensity map in the h–5–l plane measured at 0.3 K, where several intense magnetic Bragg peaks are observed. These reflections are indexed by two magnetic propagation vectors, q_{m1} = (0.17a*, 0, 0.31c*) and q_{m2} = (−0.17a*, 0, 0.31c*), related by mirror symmetry with respect to the a axis in the paramagnetic state. The magnetic origin of these peaks is confirmed by the temperature dependence of the intensity at (−1, 7, 0) – q_{m2} (**Fig. R2(b)**), which emerges below approximately 1.5 K and follows the same transition temperature as observed in bulk magnetization measurements, establishing that the reflections arise from long-range magnetic order.

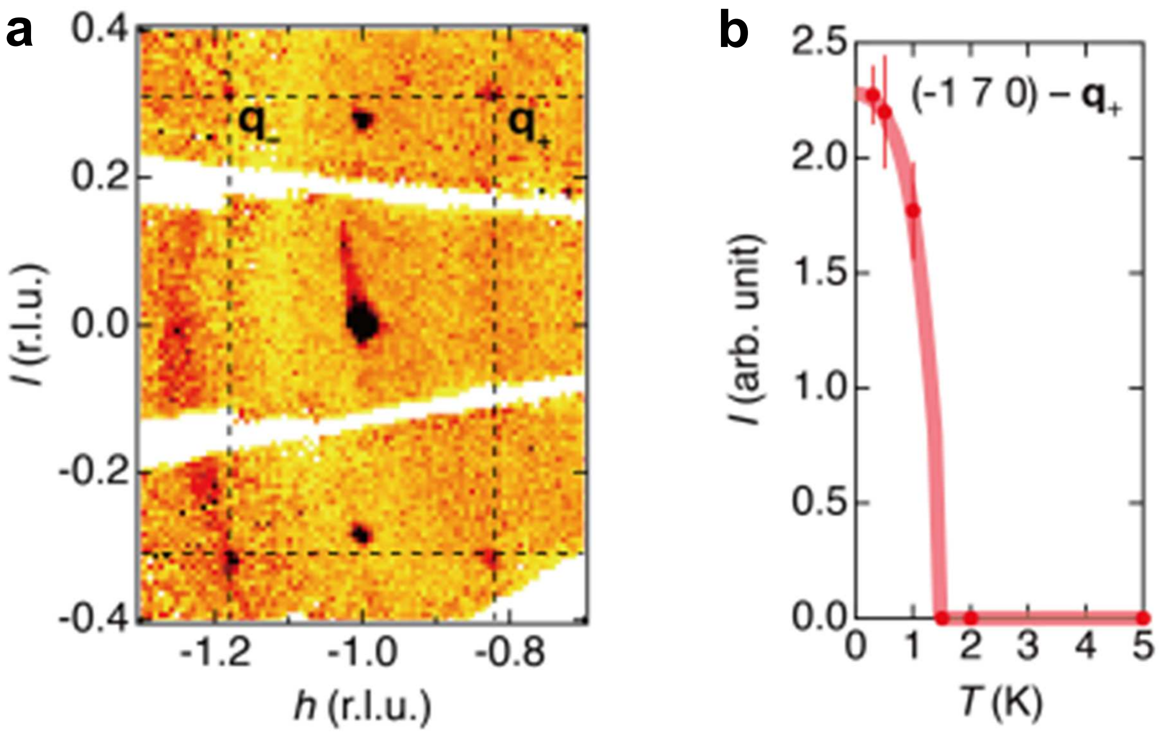

Fig. R2.

A neutron study on CeTe3 at 0.3 K and 0T.

a, Diffraction patterns in the h5l plane at 0.3 K. The magnetic peaks are noted as q_{m1} and q_{m2}.

b, Temperature dependence of the integrated intensity at (−1, 7, 0)−q_{m2}. The transparent red line is a guide to the eye. The data is taken at KEK (Japan).

REVIEWER COMMENTS

Reviewer #1 (Remarks to the Author):

The authors have made a number of significant improvements in the revised manuscript, addressing a number of the points raised after the first review round. Specifically, the original manuscript had no quasiparticle interference (QPI) data, while the revised one has QPI measurements showing Fermi surface instabilities under the in-plane magnetic field. Moreover, the revised manuscript has shown more data to ensure repeatability, as with the measurements after ramping down the field to 0 T from 2.5 T presented in Fig. S9. I am in favor of recommending this manuscript for publication if the remaining minor points are addressed:

We appreciate the reviewer's careful reading and recommendation of this manuscript for publication in Nature Communications. We have addressed the reviewer's minor points as follows:

1. The response file notes that raw dI/dV data which are not normalized by the area between 0 mV and -30 mV (as in the original manuscript), are shown in Fig. S7b. However, there is no Figure S7b. Can this be clarified? Are all the spectra shown in the manuscript now raw, or is a normalization being applied?

We apologize for the mistake in the previous response. Indeed, there should not be Figure S7.

In the revised manuscript, all spectra are presented as raw dI/dV data without normalization, to avoid arbitrary processing and to clearly validate the energy window in which the antiferromagnetic transition occurs.

2. It isn't clear from the text why the new title highlighting 'electronic moiré phases' accurately describes the impact of this work. I cannot find a description of what constitutes moiré phases in this material. Also, I cannot find the explicit connection between the charge and magnetic orders discussed in this work and the moiré phases. If these points are not discussed well, then in my humble opinion, the "moiré" part should be removed from the title and the main text.

We appreciate the reviewer's insightful comment. The central experimental result of this manuscript is the demonstration of a rich landscape of electronic (charge and spin) orderings in $CeTe_3$. In addition to the previously known CDW1, we identify CDW2mag, CDW3mag, and CBCmag, all of which can be stabilized by relatively small perturbations. Depending on the applied magnetic field, these orders can coexist or compete, giving rise to diverse combinations of propagation vectors associated with multiple charge and spin ordering states.

In our previous manuscript, we described this versatility as "versatile electronic moiré states," motivated by the general concept of moiré modulation, where superposed periodic structures generate new wave vectors through linear combinations. In that context, the term "moiré" was used in a broader, phenomenological sense to capture the coexistence of multiple electronic orders with distinct propagation vectors.

However, we recognize that this terminology may cause confusion, as "moiré" is more commonly associated with lattice twisting at small angles. To avoid ambiguity from the first stage of the manuscript, we have removed the term "moiré" from the title and abstract in the revised manuscript while preserving the central physical message. The broader perspective is retained in the Discussion section "Outlook and Future Perspectives", where it is presented as a conceptual connection rather than a strong claim. The main results and conclusions of the manuscript remain unchanged.

Detailed revisions are:
Please note that the red part highlights changes.

- The title is changed as follows

  "Versatile electronic ~~moire~~ phases enabled by intertwined multiple frustrations in antiferromagnetic two-dimensional semimetals"

- The last sentence in the abstract was changed as follows

  "Beyond establishing $CeTe_3$ as a model platform, our results demonstrate that competing instabilities in antiferromagnetic two-dimensional metals/semimetals generate versatile electronic ~~moiré~~ phases ~~beyond the paradigm of fixed moiré lattices~~, opening a route to tunable nanoscale quantum states governed by the intertwined effects of correlation, symmetry, and topology."

- The last two paragraphs, including the summary, are consistently/slightly revised to flow better.

  "Finally, guided by the general concept of moiré modulation—where the superposition of periodic structures generates new wave vectors through linear combinations—our observation of multiple electronic propagation vectors can be viewed, at a conceptual level, as a manifestation of versatile charge–spin intertwined electronic moire states, rather than a superposition tied to crystalline lattice vectors [60,61]"

  "In summary, our results establish $CeTe_3$ as a versatile platform for realizing multi-q antiferromagnetic charge orders, accompanied by large-energy-scale electronic reconstructions characteristic of strongly correlated systems. The observed phenomenology originates from the intertwining of multiple frustration channels—antiferromagnetism, charge ordering, and competing Fermi-surface instabilities—within a semimetallic state. ~~More broadly, our findings~~

~~*demonstrate that competing instabilities in antiferromagnetic two-dimensional semimetals can give rise to versatile correlated electronic moiré phases.*~~ *From a conceptual perspective, this study demonstrates that van der Waals antiferromagnetic semimetals offer promising opportunities to explore previously inaccessible, tunable quantum phases in which topology and strong electronic correlations are intrinsically intertwined in two dimensions. From a functional viewpoint, these results provide guiding principles for designing quantum materials that integrate multiple input and output channels, enabling disproportionately large electronic responses to modest external stimuli at the nanoscale in versatile electronic moire phases."*

Reviewer #2 (Remarks to the Author):

The authors have addressed my previous comments. I am happy to recommend this manuscript for publication in Nature Communications.

We greatly appreciate the reviewer's careful reading and recognizing this manuscript to be recommendable for publishing in Nature Communications.

Reviewer #3 (Remarks to the Author):

I co-reviewed this manuscript with one of the reviewers who provided the listed reports. This is part of the Nature Communications initiative to facilitate training in peer review and to provide appropriate recognition for Early Career Researchers who co-review manuscripts.

Reviewer #4 (Remarks to the Author):

The authors have successfully addressed my previous concerns by providing experimental evidence for the QPI . The inclusion of these results, along with their interpretation, has significantly improved the overall quality of the manuscript. Furthermore, the neutron scattering data has further strengthened the work. I am pleased to recommend the paper for publication in Nature Communications.

We greatly appreciate the reviewer's careful reading and recognizing this manuscript to be recommendable for publishing in Nature Communications.
